# Differentiation of Human Pluripotent Stem Cells Into Definitive Endoderm Cells in Various Flexible Three-Dimensional Cell Culture Systems: Possibilities and Limitations

**DOI:** 10.3389/fcell.2021.726499

**Published:** 2021-09-09

**Authors:** Mariia S. Bogacheva, Riina Harjumäki, Emilia Flander, Ara Taalas, Margarita A. Bystriakova, Marjo Yliperttula, Xiaoqiang Xiang, Alan W. Leung, Yan-Ru Lou

**Affiliations:** ^1^Division of Pharmaceutical Biosciences, Drug Research Program, Faculty of Pharmacy, University of Helsinki, Helsinki, Finland; ^2^Department of Clinical Pharmacy and Drug Administration, School of Pharmacy, Fudan University, Shanghai, China; ^3^Yale Stem Cell Center, Department of Genetics, Yale University, New Haven, CT, United States

**Keywords:** definitive endoderm, human pluripotent stem cell, suspension cell culture, nanofibrillar cellulose hydrogel, computational modeling, spheroid, organoid, basement membrane extract hydrogel

## Abstract

The generation of human stem cell-derived spheroids and organoids represents a major step in solving numerous medical, pharmacological, and biological challenges. Due to the advantages of three-dimensional (3D) cell culture systems and the diverse applications of human pluripotent stem cell (iPSC)-derived definitive endoderm (DE), we studied the influence of spheroid size and 3D cell culture systems on spheroid morphology and the effectiveness of DE differentiation as assessed by quantitative PCR (qPCR), flow cytometry, immunofluorescence, and computational modeling. Among the tested hydrogel-based 3D systems, we found that basement membrane extract (BME) hydrogel could not retain spheroid morphology due to dominant cell–matrix interactions. On the other hand, we found that nanofibrillar cellulose (NFC) hydrogel could maintain spheroid morphology but impeded growth factor diffusion, thereby negatively affecting cell differentiation. In contrast, suspension culture provided sufficient mass transfer and was demonstrated by protein expression assays, morphological analyses, and mathematical modeling to be superior to the hydrogel-based systems. In addition, we found that spheroid size was reversely correlated with the effectiveness of DE formation. However, spheroids of insufficient sizes failed to retain 3D morphology during differentiation in all the studied culture conditions. We hereby demonstrate how the properties of a chosen biomaterial influence the differentiation process and the importance of spheroid size control for successful human iPSC differentiation. Our study provides critical parametric information for the generation of human DE-derived, tissue-specific organoids in future studies.

## Introduction

Spheroids and organoids are three-dimensional (3D) clusters of cells. Spheroids can be made from a variety of cells including stem cells, tumor cells, and organ-specific cells. Organoids are made from stem cells or progenitors that can self-organize into organ-specific structures (Fatehullah et al., 2016; Lou and Leung, 2018). Both spheroids and organoids can produce *in vivo*–like structures and thus hold great potential in human development research, disease modeling, drug research, and tissue replacement *via* transplantation. Spheroids and organoids can be cultured and differentiated in a 3D biomaterial or in suspension without the use of a biomaterial. The selection of an appropriate biomaterial is important for successful 3D culture. Basement membrane extract (BME), such as Matrigel^TM^, is the most widely utilized biomaterial for the formation of spheroids/organoids from many cell types (Spence et al., 2011; Chua et al., 2014; Dye et al., 2015; Hohwieler et al., 2017). BME hydrogel is an animal-derived biomaterial and can interact with various types of cells *via* cell membrane receptors. For potential applications of spheroids/organoids in regenerative medicine, several xeno-free and chemically defined hydrogels have been developed (Gjorevski et al., 2016; Nowak et al., 2017; Broguiere et al., 2018; Candiello et al., 2018). Some of these systems generate spheroid/organoid-hydrogel constructs, which may cause issues in *in vivo* applications, such as hydrogel biocompatibility and biodegradation. For this reason, xeno-free hydrogels that can generate biomaterial-free cell or tissue constructs have been developed. Among them, nanofibrillar cellulose (NFC) hydrogel has been shown to support the 3D spheroid formation of human embryonic stem cells (ESCs; Lou et al., 2014), induced pluripotent stem cells (iPSCs; Lou et al., 2014), liver cancer cells HepG2 (Bhattacharya et al., 2012), and HepaRG (Malinen et al., 2014). Intact spheroids formed in NFC hydrogel can be harvested after removal of the hydrogel by utilizing a cellulase enzyme (Lou et al., 2014), thereby facilitating various downstream analyses and applications (Lou et al., 2014). NFC hydrogel displayed weak interactions with human ESCs and HepG2 cells compared with natural extracellular matrix proteins including collagens and laminins (Harjumaki et al., 2019). In contrast, suspension culture is a biomaterial-free system, usually performed in a low-adhesive culture dish or a flask for scaled-up production. Like the NFC hydrogel, suspension culture can generate scaffold-free spheroids/organoids. Suspension culture has been reported to be effective in the formation of cell aggregates and organoids (Kim et al., 2016; Bergmann et al., 2018; Kumar et al., 2019; Wimmer et al., 2019).

Although spheroid/organoid technology has been rapidly developing during the past decade, several challenges remain (Lou and Leung, 2018). One of the major challenges facing organoid technology is heterogeneity and inefficient differentiation due to uncontrolled parameters such as spheroid size and mass transfer. The heterogeneity can result in variable phenotypes and inconsistent results in downstream analyses and applications. This prompted us to study factors affecting spheroid/organoid formation. We devised the current study to investigate the 3D differentiation of human iPSCs into definitive endoderm (DE) cells using spheroids of different sizes cultured in various 3D systems. Human iPSC differentiation toward DE represents a critical step on the way to generate cell models for the liver, gut, pancreas, lungs, trachea, and thyroid (Zorn and Wells, 2009). Great strides have been made in the development of DE differentiation methods in the previous decades (D’Amour et al., 2005; Hay et al., 2008; Wang et al., 2015; Bogacheva et al., 2018). DE cells are generally characterized by the expression of specific markers: SRY-box 17 (SOX17), Cerberus 1 (CER1), hepatocyte nuclear factor 3β (HNF3B, also known as FOXA2), and chemokine receptor type 4 (CXCR4). In our previous study of DE differentiation in two-dimensional (2D) culture, we found the most efficient protocol to involve the use of activin A in a serum-free B-27-supplemented medium for 6 days (Bogacheva et al., 2018).

The current study compares three types of 3D cell culture systems: inert hydrogel-based (NFC hydrogel), cell-interacting hydrogel-based (reduced growth factor BME), and biomaterial-free (suspension) systems for their contribution to the DE differentiation of human iPSC spheroids in different sizes.

## Materials and Methods

### Cell Lines

The human iPSC line iPS(IMR90)-4 was purchased from WiCell Research Institute Inc (Madison, WI, United States), and GM23720B was purchased from Coriell Institute (United States). They were cultured on Matrigel^TM^ (BD Biosciences) with daily replenishment of the mTeSR^TM^1 medium (STEMCELL^TM^ Technologies). Subculture was performed every 4 days using Versene solution 1:5,000 (Invitrogen, 15040033) for cell detachment. Cultures were maintained at 37°C and at 5% CO_2_. Mycoplasma testing was carried out regularly by the Division of Pharmaceutical Biosciences at the University of Helsinki, Finland.

### Formation of Spheroids and SC Differentiation to DE

The human iPSCs cultured on Matrigel^TM^ in the mTeSR^TM^1 medium were dissociated into single cells by Accutase^TM^ (Millipore, SCR005). Spheroids containing a different number of cells (200, 500, and 1,000 cells per spheroid) were generated in AggreWell^TM^400 (STEMCELL^TM^ Technologies, 27845 and 34411) in the mTeSR^TM^1 medium in the presence of 10 μM Rho-associated protein kinase (ROCK)-inhibitor Y-27632 (Selleck Chemicals, S1049). After 24 h, spheroids were collected from the AggreWell^TM^400 and transferred in three different conditions for further culturing. Suspension culture condition was performed in a low-attachment 3.5-cm dish (Thermo Scientific Nunc, 174913). Hydrogel culturing was performed either in 0.55% NFC hydrogel GrowDex^®^ (UPM-Kymmene Corporation, Helsinki, Finland) in a non-adhesive 96-well plate (Corning, 3474) or in Cultrex^®^ Reduced Growth Factor BME (R&D Systems, 3533-005-02) in angiogenesis 15-well slides (ibidi, Cat# 81501, uncoated). The cell-number-to-medium-volume ratio was kept the same under all the conditions. The day when DE induction started was set as day 0. DE induction was performed for 6 days in the RPMI-1640 medium (Gibco, 31870-025) supplemented with 1× GlutaMAX^TM^ (Gibco, 35050-038), 1× B-27 (Gibco, 17504-044), 100 ng/ml activin A (PeproTech, 120-14E), and 10 μM ROCK-inhibitor Y-27632. The medium was renewed daily.

### Live/Dead Cell Staining

At the end of the differentiation experiment, spheroids cultured in BME were stained with a LIVE/DEAD^TM^ Viability/Cytotoxicity Kit for mammalian cells (Thermo Fisher Scientific, L3224) according to the instruction of the manufacturer. The dye solution consisted of Calcein AM (the final concentration was 0.5 μM) and Ethidium homodimer-1 (the final concentration was 1 μM) in the RPMI-1640 medium. The cells treated with 1% Triton X-100 for 5 min at room temperature were used as dead cell control. Spheroids were imaged within 30–60 min after staining using a confocal microscope Leica TCS SP5II HCS A with a HC PL APO 20×/0.7 CS (air) objective. Fluorescent Calcein AM (ex/em ∼495 nm/∼515 nm) produces green fluorescence in live cells. Ethidium homodimer-1 penetrates cells with damaged membranes, binds to nucleic acids, and provides red fluorescence in dead cells (ex/em ∼495 nm/∼635 nm).

### Measurement of Spheroid Diameter

The images of spheroid morphology were taken with a phase contrast microscope (Leica DM IL LED) at 5× and 10× magnifications. Spheroid diameters were then measured with the LAS EZ software (Leica Microsystems) and ImageJ (National Institutes of Health, United States).

### Collection of Spheroids From the NFC Hydrogel Culture

For downstream analyses including RNA isolation, immunofluorescence staining, and flow cytometry, spheroids were collected after removing NFC hydrogel. NFC hydrogel was removed by approximately 20-h treatment with cellulase (UPM-Kymmene Corporation, Helsinki, Finland) by following a previously described procedure (Lou et al., 2014).

### RNA Isolation and cDNA Conversion

We collected RNA samples at six time points: undifferentiated stem cells cultured in 2D (2D SC), undifferentiated stem cells in spheroids (3D SC), day 1, day 2, day 4, and day 6 of differentiation. Cells and spheroids were lysed using TRI-reagent (Zymo-research, R2050-1-50), and then RNA was isolated with a Direct-zol RNA MicroPrep kit (Zymo-research, R2060) according to the instruction of the manufacturer. The concentrations of RNA samples were measured with NanoDrop^TM^ One (Thermo Fisher Scientific). The cDNA conversion was made with a High Capacity cDNA reverse transcription kit (Applied Biosystems, 4368814) following the instructions of the manufacturer.

### Quantitative PCR

The quantitative PCR (qPCR) reactions of the obtained cDNA samples were performed on a StepOnePlus Real-Time PCR System (Applied Biosystems) using either a PowerUp SYBR Green Master Mix (Applied Biosystems, A25741) or a TaqMan^®^ Gene Expression Master Mix (Applied Biosystems, 4369016). Ribosomal protein, large, P0 (*RPLP0*) was used as a housekeeping gene. All the used primers and TaqMan Gene Expression Assay mixes are listed in Supplementary Table 1. All primers were designed by the Primer Express v2.0 software (Applied Biosystems) (Kanninen et al., 2016a) except the primers for *OCT4* (Yu et al., 2007) and *HNF3B* (D’Amour et al., 2005), and they were synthesized by Oligomer Oy (Helsinki, Finland) or Metabion (Planegg, Germany). The relative quantification of each target gene in comparison with the housekeeping gene was made by a standard curve method based on a published mathematical model (Pfaffl, 2001). The relative gene expression was calculated with reference to the undifferentiated human iPSCs in 2D culture condition.

### Immunofluorescent Staining of 2D Cell Culture

After the formation of SC spheroids in AggreWell^TM^400 for 24 h, they were collected, dissociated into single cells using Accutase^TM^ (Millipore, SCR005), suspended in the mTeSR^TM^1 medium with 10 μM ROCK-inhibitor Y-27632, and seeded on laminin-521 (LN521, Biolamina) coated black 96-well μ-plates (ibidi, 89626) to form a 2D cell monolayer. LN521 coating was prepared by incubating 10 μg/ml LN521 diluted in 1× DPBS with Ca^+^ and Mg^+^ either overnight at 4°C (slow coating) or for 2 h at 37°C (fast coating). After cells attached for 3 h, they were fixed in 4% paraformaldehyde for 10 min, permeabilized with 0.1% Triton X-100 for 10 min, and thereafter blocked with 10% normal goat or donkey serum (Millipore, Burlington, MA, United States) for 1 h. Cells were incubated with primary antibodies overnight at 4°C and then with secondary antibodies conjugated with Alexa Fluor 594 or Alexa Fluor 488 (Invitrogen) for 1 h at room temperature. Cell nuclei were stained with DAPI (Sigma-Aldrich, D8417, 12.5 μg/ml in MilliQ water) for 2 min. Primary and secondary antibodies used for immunostaining in this study are listed in Supplementary Table 2.

### Immunofluorescent Staining of 3D Spheroids

After 4 days of differentiation experiments, spheroids were collected from the culture dishes and fixed in 4% paraformaldehyde for 24 h. The next day, they were treated with 100% methanol for 2 min, then with 20% DMSO in methanol for 2 min, and again with methanol for 2 min. After that, the cells were permeabilized with 1% Triton X-100 in 1× DPBS for 2 min and then incubated in a Penetration Buffer (0.3 M Glycine + 20% DMSO + 0.2% (wt.) Triton X-100 in 1× DPBS) for 15 min with shaking. Blocking was performed with a Blocking Buffer (6% donkey serum (Southern Biotech, 0030-01) or goat serum (Gibco, 16210) + 10% DMSO + 0.2% (wt.) Triton X-100 in 1× DPBS) at 37°C for 15 min with shaking. Then the spheroids were incubated with primary antibodies diluted in an Antibody Buffer (3% donkey serum or goat serum + 5% DMSO + 0.2% Tween 20 + 10 μg/ml Heparin in 1× DPBS) at 37°C for 30 min with shaking. Thereafter, the spheroids were washed in 1× Washing Buffer (0.2% Tween 20 + 10 μg/ml Heparin in 1× DPBS) five times for 5 min each at 37°C with shaking. Then the spheroids were incubated with secondary antibody diluted in the Antibody Buffer at 37°C for 30 min with shaking followed by washing in the same way as after the primary antibody treatment. Nuclei were stained with DAPI (Sigma-Aldrich, D8417, 12.5 μg/ml in MilliQ water) for 2 min. Finally, the spheroids were treated with 100% methanol for 2 min. Visikol HISTO-M (Visikol Inc.) was added to the spheroids. Primary and secondary antibodies used for immunostaining in this study are listed in Supplementary Table 2.

### Imaging of Immunostaining

Imaging was performed on a confocal microscope Leica TCS SP5II HCS A with a HC PL APO 20×/0.7 CS (air) objective. DAPI was excited with UV (diode 405 nm/50 mW), Alexa Fluor 488 with an Argon 488 nm laser, and Alexa Fluor 594 with a DPSS (561 nm/20 mW) laser.

### Flow Cytometry

The spheroids were dissociated using Accutase^TM^ (Millipore, SCR005) at each time point as indicated. For CXCR4 and viability measurement, single cells were incubated with either PE Mouse Anti-Human CD184 (CXCR4) IgG2a (BD Biosciences, 561733) or PE Mouse IgG2a (BD Biosciences, 555574) at the concentrations according to the instruction of the manufacturer for 40 min in the dark on ice. After washing with 2% FBS (Gibco, 10270-106) in 1× DPBS, the cells were treated with 0.05 mg/ml 7-AAD Viability Staining Solution (eBioscience, 00-6993-50) for 5 min in the dark on ice. Unstained cells were used to adjust FSC, SSC, and PE-Cy5. Fluorescence compensation for the CXCR4 signal in the PE channel and the 7-AAD signal in the PE-Cy5 channel was set in an experiment using DE cells derived in 2D culture by following a previously described procedure (Bogacheva et al., 2018) and dead cells produced by the treatment with various concentrations of ethanol.

Analysis of the SSEA-4 surface marker was conducted at the same time points as for CXCR4. After treatment with Accutase^TM^, single cells were incubated with primary Mouse Anti-SSEA-4 (Developmental Studies Hybridoma Bank, MC-813-70) or Mouse IgG (PeproTech, 500-M00) at 0.2 μg/ml for 40 min on ice followed by washing with 2% FBS in 1× DPBS. Thereafter, the cells were stained with F(ab’)2-Goat Anti-Mouse-PE (eBioscience, 12-4010-82) at the concentration according to the instruction of the manufacturer for 40 min in the dark on ice.

Flow cytometric analysis was carried out on a BD LSRII flow cytometer (YellGrn Laser, with filter PE (586/15) or PE-Cy5 (670/30)) using the BD FACSDiva software. The calculation of positive cell percentage and cell viability and the visualization of the results were performed using the FlowLogic software (Inivai Technologies). An isotype control signal was used for gating the false-positive peak caused by unspecific binding. Unstained cells’ signal was used for gating living cells.

### Concentration Modeling

Nanofibrillar cellulose hydrogel-based DE differentiation was performed in 96-well plates (Corning 3474). In each well, the lower phase B contained 100 μl NFC hydrogel diluted in a medium and mixed with 3D cell spheroids (Supplementary Figure 1). The upper phase A contained a 100 μl medium supplemented with activin A whose half-life *in vivo* is 5.5 min (fast) and 20.3 min (slow) (Johnson et al., 2016). Since only phase A could be renewed daily, we used a 2× medium for phase A, which contained the RPMI-1640 medium supplemented with 2× GlutaMAX^TM^, 2× B-27, 200 ng/ml activin A, and 20 μM ROCK-inhibitor Y-27632. To model the diffusion of the key growth factor activin A from the interface between phase A and phase B into phase B, we used a computational model based on the Fick’s second law of diffusion (Berg, 1993), the general formula for half-life in exponential decay (Nelson, 2013), and the linear estimation of the diffusion constant from the literature (Bhattacharya et al., 2012).

Below is the derivation of the model. First is the Fick’s second law of diffusion written in terms of finite difference approximations to the derivatives as (Adams and Essex, 2009):

(1) $$\frac{c_{j}^{n+1}-c_{j}^{n}}{\Delta \,t}=D\,\frac{c_{j+1}^{n}-2\,c_{j}^{n}+c_{j-1}^{n}}{\Delta \,x^{2}}$$

where *c* is the concentration at time point *n* = {0,Δ*t*,2Δ*t*,…*N*Δ*t*}, at position *j* = {0,Δ*x*,2Δ*x*,…*J*Δ*x*}, and *D* is the diffusion constant. By defining the constant = $\frac{D\,\Delta \,t}{\Delta \,x^{2}}$, Equation 1 can be expressed as:

(2) $$c_{j}^{n+1}=S\,\left(c_{j+1}^{n}+c_{j-1}^{n}\right)+\left(1-2\,S\right)\,c_{j}^{n}$$

Denaturation of protein growth factors is considered by including the general formula for exponential degradation, with half-life λ for time step Δ*t*:

(3) $$c_{j}^{n+1}=\left[S\,\left(c_{j+1}^{n}+c_{j-1}^{n}\right)+\left(1-2\,S\right)\,c_{j}^{n}\right]\,{\left(\frac{1}{2}\right)}^{\Delta \,t/\lambda }$$

By combining the Einstein relation of kinetic theory with the definition of the viscous friction coefficient (Nelson, 2013), the diffusion coefficient can be expressed in terms of the Boltzmann constant *k_B*, temperature *T*, time step Δ*t*, and mass *m*:

(4) $$D=\frac{k_{B}\,T\,\Delta \,t}{2\,m}$$

A previous study (Bhattacharya et al., 2012) had measured the diffusion constants of Dextran with different masses in NFC hydrogel. Since we can assume that the temperature of the hydrogel had been constant in each study for Dextran diffusion, we can consider *T* as a constant in the above equation. As all the other terms are constants, we can infer that the diffusion constant *D* and the mass of the diffusing particle *m* have an inverse linear correlation. Derived from the publication (Bhattacharya et al., 2012) *via* linear regression (*R*^2^0.999), the function takes the form:

(5) $$D=1.76\cdot 10^{-7}\,\frac{1}{m}+1.44\cdot 10^{-5}$$

With these definitions, the diffusion constant *D* could be estimated for an object of mass *m* in NFC hydrogel. Diffusion was modeled based on Equation 3, with the diffusion constant estimated as in Equation 5 by using MatLab R2014a (8.3.0.532).

### Statistical Analysis

Statistical analyses were performed using the GraphPad Prism 8 software. Statistical significance was determined by one-way analysis of variance (ANOVA) followed by Sidak’s multiple comparisons test or Tukey’s multiple comparisons test as recommended by the software. Kruskal–Wallis test followed by Dunn’s multiple comparisons test was used in case a one-way ANOVA test could not be performed because the data did not pass a normality test. Correlation analysis was performed using a Pearson’s correlation test. Differences of adjusted *p* < 0.05 (^∗^), adjusted *p* < 0.01 (^∗∗^), adjusted *p* < 0.001 (^∗∗∗^), and adjusted *p* < 0.0001 (^****^) were considered significant.

## Results

### Initial Spheroid Size (Cell Number) Affects Spheroid Survival During DE Differentiation

In subconfluent 2D cell culture where cells are well spread into monolayers, soluble factors in differentiation media are accessible to every cell. In contrast, in 3D cell culture, only cells in the outermost layer of spheroids/organoids are directly accessible by media components, but cells apart from the outermost layer must rely on other means such as diffusion or active transport in order to gain access to the media. To study the influence of cell layer thickness during cell differentiation, we generated spheroids using five different initial cell numbers (50, 100, 200, 500, and 1,000 cells per spheroid) using AggreWell^TM^ 400 plates that contain microfabricated wells for the formation of homogenous spheroids. The initial size of each formed spheroid can be controlled by adjusting the input cell number, which requires the dissociation of stem cell colonies into single cells. This procedure results in the breakage of cell–cell interactions and cell–membrane junctions. The consequence of these events is the change in the balance between the actin-myosin anchoring force and the contraction force. The predominance of the contraction force stimulates cell death in the case of failure on re-adhesion (Chen et al., 2010). Rho-associated protein kinase (ROCK) is involved in the process of actin-myosin contraction. Treatment with the ROCK inhibitor Y-27632 improves stem cell viability in single cell status (Watanabe et al., 2007). Prior to the spheroid formation, we treated single human iPSCs iPS(IMR90)-4 cells with Y-27632 to prevent cell death. We initially chose two 3D culture conditions, suspension (without biomaterial) and NFC hydrogel. To differentiate human iPSCs into DE cells, we adopted an activin A-based protocol that was shown to be the most effective under 2D cell culture condition (Bogacheva et al., 2018). After 24 h in AggreWell^TM^ 400 plates, the formed spheroids were transferred either into NFC hydrogel or into suspension in a differentiation medium supplemented with Y-27632. Y-27632 was used up to day 2 of differentiation.

**FIGURE 1:**
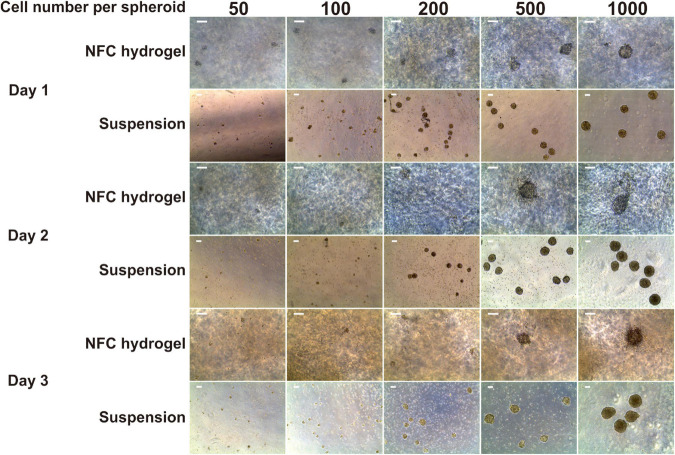
Morphology of iPS(IMR90)-4-derived spheroids in five sizes: 50, 100, 200, 500, and 1,000 cells per spheroid cultured in suspension and nanofibrillar cellulose (NFC) hydrogel. Days 1, 2, and 3 of definitive endoderm (DE) differentiation are presented. The cell number in spheroids was controlled using the AggreWell400 plate for 24-h spheroid formation. Afterward, spheroids were collected from AggreWell400 and seeded in either low-adhesive Petri dishes in suspension or in NFC hydrogel in low-adhesive 96-well plates. Scale bars = 100 μm.

We obtained only a few aggregates with the initial cell number of 50 and 100 cells per spheroid in both suspension and NFC hydrogel cultures (Figure 1). Most of the survived aggregates did not acquire a round shape and were surrounded by detached cells at day 1. Subsequently, all of them dissociated within 2 days. Due to the low survival rate of spheroids with the initial cell number of 50 and 100 cells per spheroid, we excluded them from further studies.

Spheroids with the initial cell number of 200 cells survived better in suspension culture than in NFC hydrogel with spheroid yield in suspension culture being 5.5-fold more than in NFC hydrogel at day 3. Spheroids with the initial cell number of 500 and 1,000 cells survived well in both conditions (Figure 1). They remained round by day 3. The DE differentiation medium can induce cell death as observed in our previous 2D DE differentiation study (Bogacheva et al., 2018). As expected, we also noticed dead cells in the 3D cultures. This phenomenon was more obvious around spheroids in NFC hydrogel culture probably because dead cells were more physically restricted within the hydrogel environment.

### ROCK Inhibitor Y-27632 Improves Spheroid Survival During DE Differentiation

**FIGURE 2:**
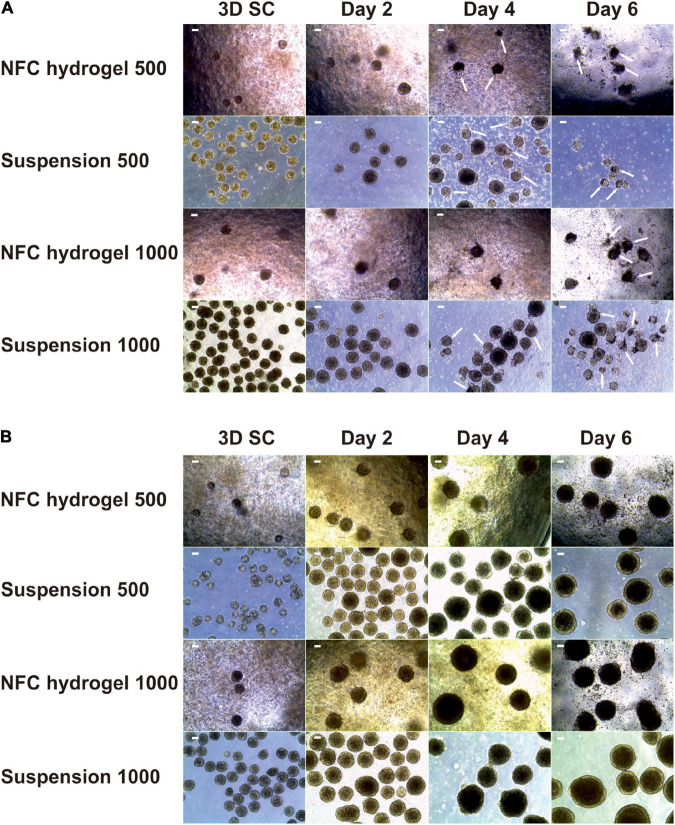
Morphology of iPS(IMR90)-4 cell spheroids with the initial cell number of 500 and 1,000 cells per spheroid at the undifferentiated stage (3D SC) and at days 2, 4, and 6 of DE differentiation in suspension and NFC hydrogel. **(A)** Spheroids treated with a 10 μM Rho-associated protein kinase (ROCK) inhibitor Y-27632 up to day 2 of differentiation procedure. Arrows show some of damaged spheroids. Scale bars = 100 μm. **(B)** Spheroids treated with 10 μM Y-27632 throughout the entire differentiation experiment. Scale bars = 100 μm.

Initially, we treated iPS(IMR90)-4-derived spheroids with Y-27632 up to day 2 of differentiation but observed massive cell death (Figure 2A, single cells between spheroids) and loss of spheroid shape (Figure 2A, arrows) from day 4 onward. To obtain higher cell viability, we used Y-27632 in differentiation media for the entire period of the experiment (Figure 2B). In the presence of Y-27632, spheroids were well maintained during the 6-day differentiation. To investigate whether Y-27632 may have any negative effect on DE differentiation, we studied its effect on gene expression in iPS(IMR90)-4 cells cultured under conventional 2D condition. Undifferentiated stem cells grown in colonies were dissociated with Accutase^TM^ and were cultured in the mTeSR^TM^1 medium supplemented with Y-27632 for the first day or for 7 days (Supplementary Figure 2A). There were no statistically significant changes in the mRNA expression of all the studied genes in 1-day treated cells compared with day 0 cells (Supplementary Figure 2B). Treatment with Y-27632 for 7 days did not significantly change the gene expression of *OCT4*, *HNF3B*, *CXCR4*, *BRACHYURY*, and *SOX1* (Supplementary Figure 2B). However, it significantly increased the mRNA expression of *NANOG* by 2.3-folds (adjusted *p* = 0.0327) and *CER1* by 4.2-folds (adjusted *p* = 0.0028). Earlier studies have shown the increased expression of *NANOG* during DE differentiation in 2D culture (Bogacheva et al., 2018), and NANOG was shown to be involved in early differentiation, for example, by participating in DE specification and repressing embryonic ectoderm differentiation (Teo et al., 2011; Wang et al., 2012). The upregulation of the specific DE marker *CER1* is a strong indication for DE differentiation. Therefore, we chose to use Y-27632 during the entire differentiation period.

### 3D Conditions Influence Spheroid Morphology During DE Differentiation

Based on the results from the 2D DE differentiation experiments showing that 6-day DE induction is the most effective method (Bogacheva et al., 2018), we performed 6-day DE differentiation of iPS(IMR90)-4 cells in three 3D conditions. Suspension culture in this study represents a biomaterial/scaffold-free 3D culture condition, while NFC hydrogel is an inert biomaterial that exhibits weak nonspecific interactions with cells (Harjumaki et al., 2019). Conversely, BME interacts with stem cells and DE cells directly *via* cell membrane receptors, so it represents an active cell-interacting biomaterial. We differentiated spheroids with the initial cell number of 500 and 1,000 cells in reduced growth factor BME and 200, 500, and 1,000 cells per spheroid in suspension or NFC hydrogel. Y-27632 was used throughout the entire experiment. At day 0, spheroids under all three culture conditions were transparent and round (Supplementary Figure 3). Hollow structures were seen in some of the spheroids at day 0, but these structures disappeared from day 2 onward. Spheroids in BME started to disintegrate and to lose their typical spherical shape morphology from day 2 onward as a result of cell migration out of the spheroids, thereby turning part of 3D culture to resemble 2D culture (Supplementary Figures 3E,H, arrows). On the contrary, spheroids in both suspension and NFC hydrogel retained a clear spherical shape during the entire differentiation experiment (Supplementary Figures 3A–D,F,G). Some larger spheroids in suspension and NFC hydrogel became darker in the center after day 2. At day 6, we observed an increase in the spheroid size and condensed darker area in the center of spheroids grown in both NFC hydrogel and in suspension. From day 4 to day 6, cells that had migrated out of the spheroids in BME started to die, as seen by cell morphology and live/dead staining (Supplementary Figure 3). Since BME did not support the morphology of the spheroids, we excluded the BME condition from this study.

### Changes in Spheroid Size (Diameter) During DE Differentiation

**FIGURE 3:**
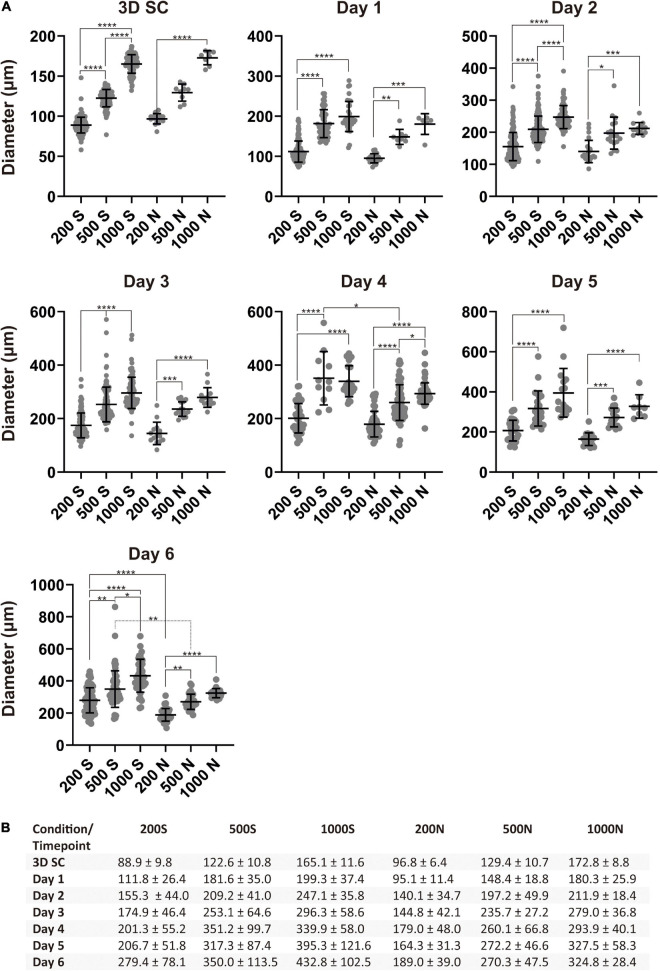
Spheroid sizes during differentiation. **(A)** Size distribution of iPS(IMR90)-4 cell spheroids with the initial cell number of 200, 500, and 1,000 cells per spheroid during DE differentiation in suspension (S) and NFC hydrogel (N). Diameters (μm) were measured daily during the experiment. Horizontal lines are mean values, and vertical lines are SD. Because a normality test indicates that the data were not sampled from a Gaussian population, Kruskal–Wallis test followed by Dunn’s multiple comparisons test was used. Statistical significance * adjusted *p* < 0.05, ** adjusted *p* < 0.01, *** adjusted *p* < 0.001, and **** adjusted *p* < 0.0001 are shown above lines. **(B)** Average diameter of spheroids with the initial cell number of 200, 500, and 1,000 cells per spheroid (μm) ± SD at each day of the DE differentiation of iPS(IMR90)-4 cells.

After excluding BME from the study, we chose suspension and NFC hydrogel conditions in the following experiments. We differentiated iPS(IMR90)-4 spheroids with the initial cell number of 200, 500, and 1,000 cells per spheroid in suspension, named 200S, 500S, and 1000S, and in NFC hydrogel, named 200N, 500N, and 1000N, respectively. We monitored the diameters of spheroids during the 6-day differentiation (Figure 3). Spheroids under all the conditions gradually increased in size every day as measured by their diameters (Figure 3B and Supplementary Figure 4). Significant diameter increase day by day was seen more frequently in suspension culture than in NFC hydrogel culture (Supplementary Figure 4). At day 0, undifferentiated 3D SC spheroids under the conditions 200S, 500S, and 1000S were significantly different in size (adjusted *p* < 0.0001, Figure 3A), whereas in NFC hydrogel, a significant difference in size was only detected between 200N and 1000N (adjusted *p* < 0.0001, Figure 3A). At day 1, 200S or 200N spheroids were significantly different from 500S/500N to 1000S/1000N spheroids in both suspension and NFC hydrogel (Figure 3A). At days 2 and 3, we found statistically significant differences between the spheroids of all sizes in suspension (adjusted *p* < 0.0001), but the difference in NFC hydrogel was similar to that at day 1 (Figure 3A). At day 4, we did not observe a significant difference between 500S and 1000S, but we found a significant difference between 200S and 500S, as well as between 200S and 1000S. In NFC hydrogel, a significant difference was detected among all the conditions at day 4 (Figure 3A). Moreover, at day 4, 500S spheroids had significantly greater diameters than 500N (adjusted *p* = 0.0452). At day 5, 200S or 200N spheroids were significantly different from 500S/500N to 1000S/1000N spheroids in both suspension and NFC hydrogel. At day 6, spheroids in suspension were all different, while in NFC hydrogel, the difference remained only between 200N and 500N, as well as between 200N and 1000N. In addition, 200S and 500S were bigger than 200N and 500N, respectively (Figure 3A).

In summary, we found that 200S/200N spheroids were significantly smaller than 1000S/1000N spheroids in both suspension and NFC hydrogel during the entire differentiation experiment. However, we did not detect a significant difference between 500N and 1000N spheroids in NFC hydrogel at most days except at day 4. Therefore, we decided to compare 200S and 200N with 1000S and 1000N spheroids in terms of the effectiveness of DE differentiation in the subsequent immunostaining and flow cytometry experiments.

We repeated this experiment using another human iPSC line GM23720B. We differentiated GM23720B spheroids with the initial cell number of 200 and 1,000 cells per spheroid in suspension and NFC hydrogel during 4 days and assessed the dynamics of their diameter changes (Supplementary Figures 5, 6). Similar to iPS(IMR90)-4, GM23720B spheroids have gradually increased in diameters during the differentiation (Supplementary Figures 5B, 6). Also, a significant diameter increase day by day was seen more frequently in suspension culture than in NFC hydrogel culture (Supplementary Figure 6). Undifferentiated 3D SC spheroids containing 200 and 1,000 cells were significantly different in size in both suspension and NFC hydrogel (adjusted *p* < 0.0001, Supplementary Figure 5A). In suspension, the difference between 200S and 1000S spheroids remained until day 4 (adjusted *p* < 0.0001, Supplementary Figure 5A). In NFC hydrogel, 200N were significantly smaller in diameter than 1000N until day 2 (adjusted *p* < 0.0001, Supplementary Figure 5A). At day 4, a significant difference between 200N and 1000N was detected again (adjusted *p* < 0.05, Supplementary Figure 5A). At day 3 and day 4, the diameter of 1000S spheroids was significantly bigger than 1000N (adjusted *p* < 0.05, Supplementary Figure 5A).

### Gene Expression Profiles in iPS(IMR90)-4 Cells and Their Derivatives During DE Differentiation in 3D Conditions

**FIGURE 4:**
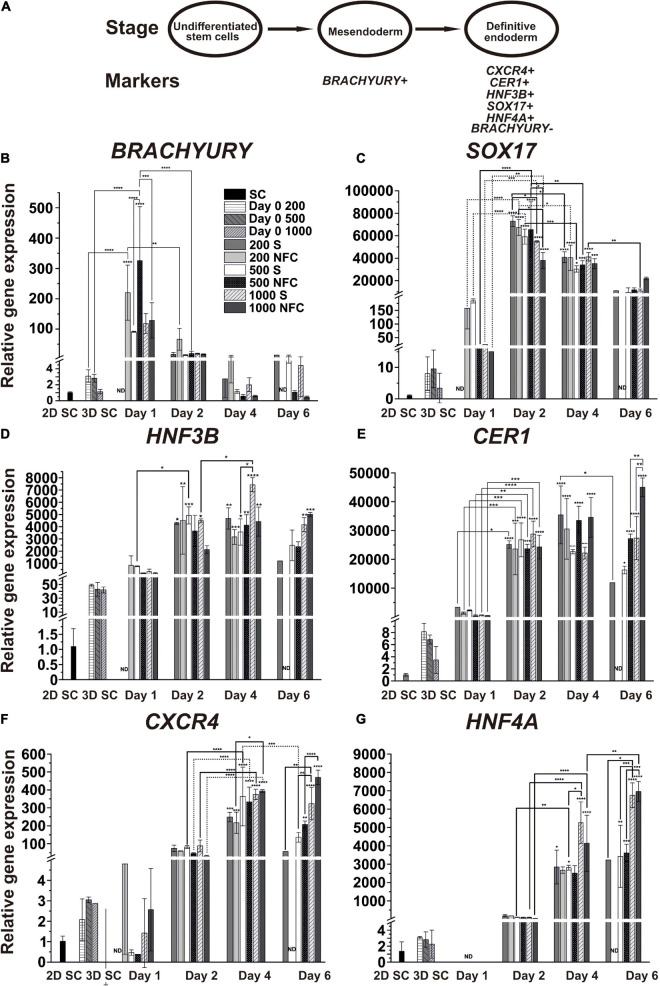
**(A)** Schematic of the characteristic markers at the studied differentiation stages. **(B–G)** The mRNA expression patterns of the mesendoderm (*BRACHYURY*), DE (*SOX17*, *HNF3B*, *CER1*, and *CXCR4*), and hepatic endoderm (*HNF4A*) specific markers during DE differentiation of iPS(IMR90)-4 cells in 3D spheroids with the initial cell number of 200, 500, and 1000 cells per spheroid in suspension (S) and NFC hydrogel (N). Relative gene expression was measured by qPCR and normalized with RPLP0 housekeeping gene. Fold inductions were calculated with the reference to the stem cell samples (2D SC). *N* = 2 or 3 biological repeats. ND, no data. Error bars are SD. One-way ANOVA followed by Tukey’s multiple comparisons test was used to compare between any pairs. Statistical significance *adjusted *P* < 0.05, **adjusted *P* < 0.01, ***adjusted *P* < 0.001, and ****adjusted *P* < 0.0001 in comparison with 2D SC are shown above bars. Statistically significant differences *adjusted *P* < 0.05, **adjusted *P* < 0.01, ***adjusted *P* < 0.001, and ****adjusted *P* < 0.0001 between days 1 and 2, days 2 and 4, and days 4, and 6 are shown above lines. Breaks in *Y*-axis in the figure panels are *BRACHYURY*: 5–7, *SOX17*: 16–26 and 200–9000, *HNF3B*: 2–5 and 60–150, *CER1*: 10–50, *CXCR4*: 5–30, and *HNF4A*: 5–30.

To study how spheroid size and 3D culture condition affect DE differentiation, we analyzed the gene expression profiles of mesendoderm, DE, and hepatic endoderm specific markers (Figure 4A) in 200S, 200N, 500S, 500N, 1000S, and 1000N at day 0, day 1, day 2, day 4, and day 6 of the differentiation experiment. Mesendoderm is a progenitor cell stage established prior to DE specification when differentiating PSCs still retain developmental plasticity to generate either mesoderm or DE.

We first validated our approach in DE differentiation by normalizing the transcript expression levels of individual genes to their highest level during DE differentiation (Supplementary Figure 7). We confirmed that the induction of these genes followed the sequence *BRACHYURY* (day 1) → *SOX17* (day 2) → *HNF3B*/*CER1* (day 2-4) → *CXCR4* (day 4) → *HNF4A* (day 6), which was also previously observed from other DE differentiation studies (D’Amour et al., 2005; McLean et al., 2007; Teo et al., 2012).

Next, we examined the expression of individual mesendoderm and endoderm genes according to their relative expression levels to 2D SC controls, which represent undifferentiated human iPSCs, to reveal differences among conditions for different initial cell numbers and different 3D culture strategies, namely NFC hydrogel versus suspension cultures (Figures 4B–G). *BRACHYURY* (*BRA* or *TBXT*) is a specific marker for mesendoderm lineage cells, and its expression turns off once cells become specified as DE. As expected, we observed a strong upregulation of its expression at day 1 in 200N and 500N cells, followed by downregulation on the next day (Figure 4B). Its expression stayed low or further reduced at day 4 and day 6, corroborating that by day 4, the cells were specified as DE under all the conditions.

The gene expression of the DE marker *SOX17* was significantly higher in the cells at day 2 and day 4 in all the conditions in comparison with the stem cells and the cells at day 1 of differentiation (Figure 4C). At day 2, 200S and 200N cells had higher *SOX17* gene expression than 1000S (adjusted *p* = 0.034) and 1000N cells (adjusted *p* = 0.0123), respectively. 500N cells also showed a higher *SOX17* level than 1000N cells (adjusted *p* = 0.0271). At day 4, *SOX17* expression significantly dropped in 200S (adjusted *p* = 0.0128), 200N (adjusted *p* = 0.0374), 500S (adjusted *p* = 0.0002), and 500N cells (adjusted *p* = 0.0055) compared to the day 2 level. By day 6, all the conditions did not exhibit a significant difference in *SOX17* expression compared with the undifferentiated stem cells, and 1000S cells even had significantly lower expression than that at day 4 (adjusted *p* = 0.0016).

The gene expression of the DE marker *HNF3B* significantly increased at day 2 in all suspension conditions and 200N except 500N and 1000N, but by day 4, it increased in all the conditions (Figure 4D). It was significantly upregulated in 1000S cultures by day 4 compared with day 2 (adjusted *p* = 0.0161). 1000S cultures at day 4 also had higher *HNF3B* expression than 500S cultures (adjusted *p* = 0.016). There was no significant increase from day 4 to day 6. The expression of *HNF3B* in 200S, 500S, and 500N cells at day 6 was not significantly different in comparison with the undifferentiated stem cells.

The gene expression of another DE marker *CER1* (Iwashita et al., 2013) significantly increased in all six conditions at day 2 when compared with the stem cells and the cells at day 1 (Figure 4E). On the following days, *CER1* expression did not increase significantly or even dropped in 200S cells at day 6 in comparison with day 4 (adjusted *p* = 0.0209). 1000N cells at day 6 had higher *CER1* expression than 500N (adjusted *p* = 0.0038) and 1000S cells (adjusted *p* = 0.0044).

CXCR4 is a well-characterized DE marker (D’Amour et al., 2005). Its expression was significantly upregulated under all the conditions at day 4 (Figure 4F). By day 6, it remained at the same level in 500N, 1000S, and 1000N cells and decreased in 500S cells. 1000N spheroids at day 4 showed higher *CXCR4* expression than 200N spheroids (adjusted *p* = 0.016). 1000S spheroids at day 6 displayed higher *CXCR4* expression than 200S (adjusted *p* = 0.0073) and 500S spheroids (adjusted *p* = 0.0079). Similarly, 1000N spheroids had higher *CXCR4* expression than 500N cells (adjusted *p* < 0.0001).

HNF4A isoforms are differentially expressed during development. HNF4A 1D isoform (transcribed from the P2 promoter) was earlier shown to increase in DE cells and promote DE differentiation (Hanawa et al., 2017). In the current study, we measured total *HNF4A* isoforms and found that their expression significantly increased at day 4 in 200S, 500S, 1000S, and 1000N cells, whereas 1000S cells had higher *HNF4A* expression than 500S cells (adjusted *p* = 0.0217; Figure 4G). By day 6, *HNF4A* increased in 1000N cells compared to the day 4 level (adjusted *p* = 0.0039). 1000S cells at day 6 showed higher *HNF4A* expression than 200S (adjusted *p* = 0.0179) and 500S cells (adjusted *p* = 0.0004). 1000N cells had higher *HNF4A* expression than 500N cells (adjusted *p* = 0.0003).

Taken together, we found that the studied DE markers were significantly increased at day 2 or day 4 in comparison with the undifferentiated stem cells. Only *HNF4A* expression was further increased from day 4 to day 6. Intriguingly, the expression of the DE markers *SOX17*, *CER1*, and *CXCR4* started to decrease at day 6 suggesting an accelerated differentiation program compared with 2D adherent differentiation (Bogacheva et al., 2018). Hence, we selected the 4-day differentiation protocol in the following experiments. Since the PCR method determines gene expression in bulk cell populations, we next used flow cytometry and immunofluorescence staining to assess DE differentiation in individual cells.

### Spheroid Size and 3D Culture Conditions Affect the Efficiency and the Effectiveness of DE Formation

Before DE differentiation, we stained pluripotency markers in iPS(IMR90)-4 spheroids after their formation in AggreWell^TM^ 400 plates. All the spheroids with 50, 100, 200, 500, and 1,000 cells per spheroid were positive for OCT4 and NANOG proteins (Supplementary Figure 8).

**FIGURE 5:**
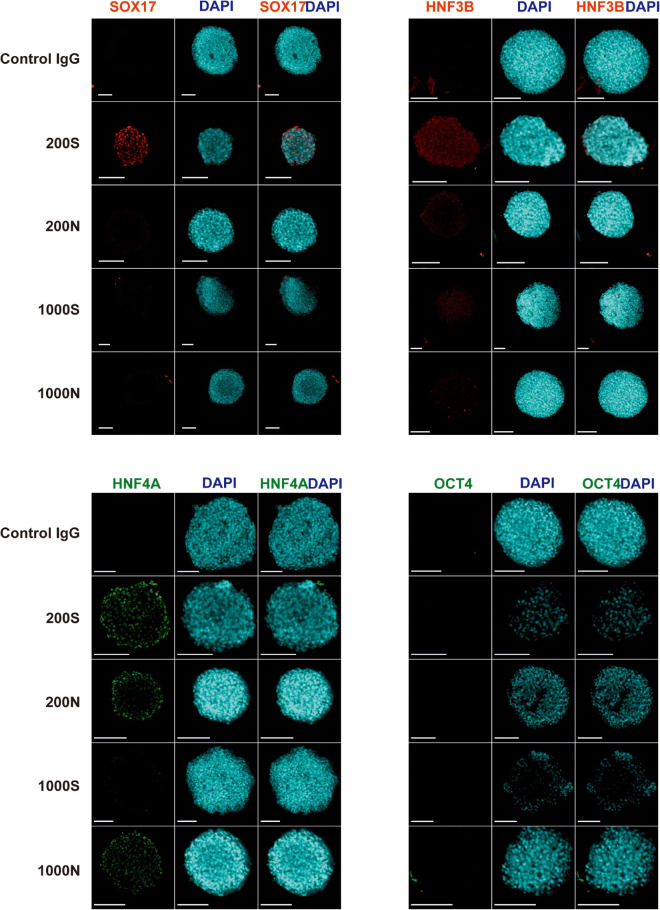
Expression of SOX17, HNF3B, HNF4A, and OCT4 proteins in iPS(IMR90)-4 cell spheroids at day 4 of 3D DE differentiation in suspension (S) or NFC hydrogel (N). Nuclei of cells were stained with DAPI (blue). Proteins of interest were stained either with Alexa Fluor 488 (HNF4A and OCT4), showed in green, or with Alexa Fluor 594 (SOX17 and HNF3B), showed in red. Scale bars = 100 μm.

We evaluated the efficiency and the effectiveness of the DE formation by image-based analysis of SOX17, HNF3B, and HNF4A protein expression and quantitative analysis of CXCR4 expression. We conducted immunofluorescence staining for the DE markers SOX17, HNF3B, and HNF4A and the pluripotency marker OCT4 in day 4 spheroids (Figure 5 and Supplementary Figure 9). 200S iPS(IMR90)-4 spheroids were positive for SOX17, HNF3B, and HNF4A (Figure 5). 200N spheroids were strongly positive for HNF4A protein and weakly so for HNF3B in the cells of the outer layers (Figure 5), which displayed similar intensity to what was observed in 2D DE differentiation previously (Bogacheva et al., 2018), but 200N spheroids were negative for SOX17 (Figure 5). 1000S spheroids had the weakly positive expression of HNF3B and HNF4A proteins and did not express SOX17 (Figure 5). 1000N spheroids showed strong HNF4A expression and were negative for SOX17 and HNF3B (Figure 5). None of the conditions showed OCT4 protein expression (Figure 5).

Similar to iPS(IMR90)-4 DE spheroids, OCT4 protein expression was not observed in day 4 GM23720B cell spheroids in all the conditions (Supplementary Figure 9). 200S spheroids were positive for SOX17 and HNF3B proteins while negative for HNF4A (Supplementary Figure 9). 200N and 1000S spheroids had positive signals for SOX17 (Supplementary Figure 9). 1000N spheroids demonstrated low or no expression for the stained protein markers (Supplementary Figure 9).

**FIGURE 6:**
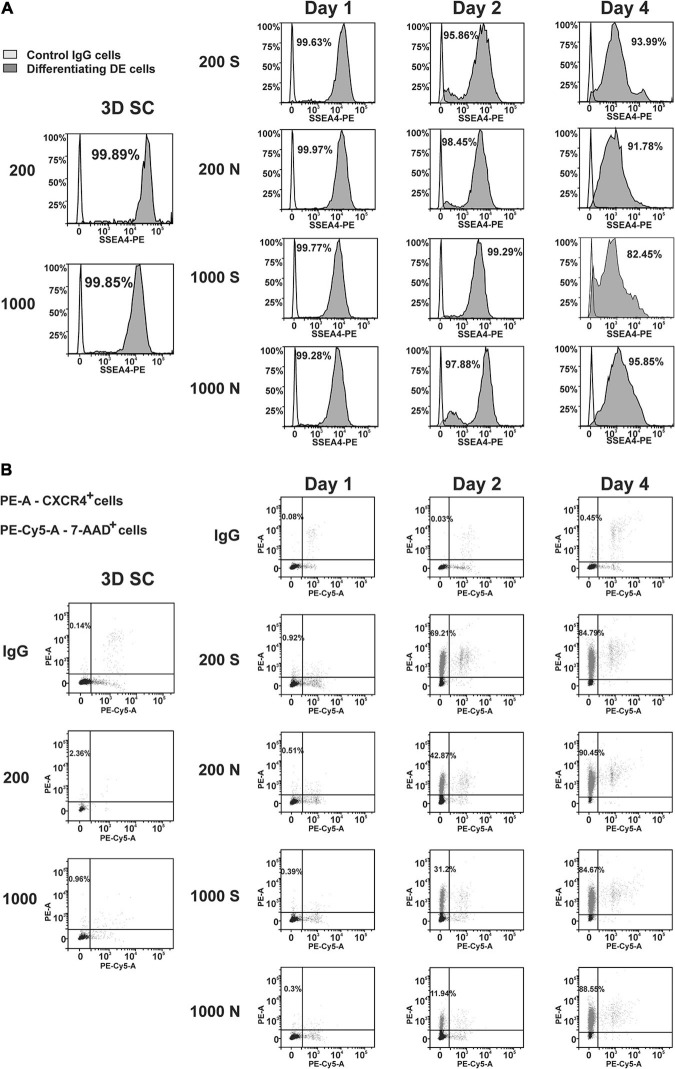
Dynamic expression of the pluripotency (SSEA4) and DE (CXCR4) protein markers in iPS(IMR90)-4 cells and their derivatives during DE differentiation of 3D spheroids with the initial cell number of 200 and 1,000 cells per spheroid in suspension (S) and NFC hydrogel (N). Graphic results from one out of four biological repeats are presented in the figure. Graphics are generated using the Flowlogic software (Inivai Technologies). **(A)** SSEA4 protein expression at the stage of undifferentiated cells (3D SC) and day 1, day 2, and day 4 in differentiation experiments, in which the same number of spheroids was harvested from suspension (S) and NFC hydrogel (N). Cells stained with an SSEA4 antibody or normal IgG were measured in the PE channel by flow cytometry. Gating was done based on IgG control as SSEA4^–^. Percentage on each histogram means a percent of SSEA4^+^ cells. **(B)** CXCR4 protein expression at the stage of undifferentiated cells (3D SC) and day 1, day 2, and day 4 in differentiation experiments, in which the same number of spheroids was harvested from suspension (S) and NFC hydrogel (N). The percentage of CXCR4^+^ cells was calculated by flow cytometry analysis. Cells were stained with PE mouse anti-human CD184 (CXCR4) IgG2a or PE mouse IgG2a that was detected in the PE channel and then with 7-AAD Viability Staining Solution that was detected in the PE-Cy5 channel. The percentage of live CXCR4^+^ cells is presented in the upper left quadrant of each dot-blot. This experiment was performed four times, and only one of the experimental results is shown here.

**FIGURE 7:**
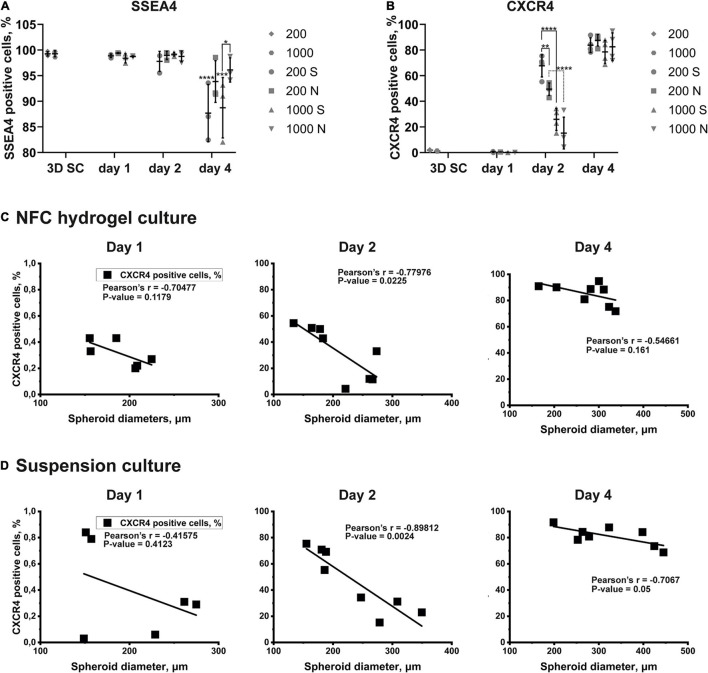
The quantitative expression of markers and the correlation between spheroid diameters and the effectiveness of the DE differentiation in iPS(IMR90)-4 cells. **(A)** SSEA4 protein expression pattern in iPS(IMR90)-4 cell spheroids (3D SC) and their derivatives during 3D DE differentiation in suspension (S) and NFC hydrogel (N). *N* = 3 biological repeats. Data were analyzed by one-way ANOVA followed by Sidak’s multiple comparisons test. Statistical significance *** adjusted *p* < 0.001 and **** adjusted *p* < 0.0001 in comparison with 3D SC are shown above scatters. Statistically significant difference * adjusted *p* < 0.05 between conditions 1000S and 1000N at day 4 is shown above the line. **(B)** CXCR4 protein expression pattern in iPS(IMR90)-4 cell spheroids (3D SC) and their derivatives during 3D DE differentiation in suspension (S) and NFC hydrogel (N). *N* = 4 biological repeats. One-way ANOVA followed by Sidak’s multiple comparisons test was used. Statistically significant differences ** adjusted *p* < 0.01 and **** adjusted *p* < 0.0001 between conditions are shown above lines. **(C)** Pearson correlation between spheroid diameters and percentages of live CXCR4^+^ cells during 3D DE differentiation in NFC hydrogel. Statistically significant negative Pearson correlation is detected at day 2. **(D)** Pearson correlation between spheroid diameters and percentages of live CXCR4^+^ cells during 3D DE differentiation in suspension. Statistically significant negative Pearson correlation is detected at day 2.

To quantify the differentiation in individual cells, we performed flow cytometry analysis of iPS(IMR90)-4 and GM23720B spheroids and their derivatives differentiated in suspension or NFC hydrogel at day 1, day 2, and day 4 of DE differentiation (Supplementary Figure 10). We examined the pluripotency marker SSEA4 and the DE marker CXCR4 in iPS(IMR90)-4 spheroids (Figures 6, 7, and Supplementary Figure 11) and the DE marker CXCR4 in GM23720B spheroids (Supplementary Figure 12).

The percentage of SSEA4^+^ cells in iPS(IMR90)-4 cell spheroids before differentiation was more than 99% (Figures 6A, 7A), and it significantly dropped by day 4 only in spheroids cultured in suspension (adjusted *p* < 0.0001 for 200S and adjusted *p* = 0.0003 for 1000S, Figure 7A). We also observed that the fluorescent intensity peaks in all histograms shift to the left during the differentiation (Figure 6A and Supplementary Figure 11). At day 4, 1000S spheroids had significantly less SSEA4^+^ cells than 1000N spheroids (adjusted *p* = 0.0139, Figure 7A).

Spheroids with the initial cell number of 200 and 1,000 cells per spheroid at the stem cell stage were negative for CXCR4 protein (Figures 6B, 7B, and Supplemetary Figure 12A). The percentage of CXCR4^+^ iPS(IMR90)-4 cells did not change at day 1 but rose at day 2 and then reached its maximum by day 4 (Figures 6B, 7B). Similarly, the percentage of CXCR4^+^ GM23720B cells also increased at day 2 and day 4 (Supplemetary Figure 12A). At day 2, 200S iPS(IMR90)-4 spheroids displayed the highest percentage of CXCR4^+^ cells (67.7 ± 8.6%, Figure 7B). This percentage was significantly higher than that for 200N (49.5 ± 4.9%, adjusted *P* = 0.0056) and 1000S spheroids (25.9 ± 8.6%, adjusted *P* < 0.0001, Figure 7B). Similarly, 200S GM23720B spheroids also displayed higher percentage of CXCR4^+^ cells (62.7 ± 5.8%) than that for 200N (54.0 ± 5.0%) and 1000S spheroids (55.8 ± 5.5%, Supplemetary Figure 12A). For the iPS(IMR90)-4 spheroids in NFC hydrogel, a similar trend to suspension cultures was observed: 200N spheroids (49.5 ± 4.9%) displayed a significantly higher percentage of CXCR4^+^ cells than 1000N spheroids (15.2 ± 12.4%, adjusted *p* < 0.0001, Figure 7B).

Furthermore, we found a negative correlation between iPS(IMR90)-4 spheroids’ size and percentage of CXCR4^+^ cells in both NFC hydrogel (*p* = 0.0225, Figure 7C) and suspension (*p* = 0.0024, Figure 7D) at day 2 of the differentiation. For GM23720B spheroids, the correlation was found between spheroids’ size and percentage of CXCR4^+^ cells in suspension at day 4 of the differentiation (*p* = 0.0251, Supplementary Figure 12C).

When measuring the CXCR4 expression by flow cytometry, we also stained the cells with 7-AAD fluorescent dye to assess cell death in spheroids. We did not detect a significant difference in cell death between time points or culture conditions (Supplementary Figure 13). The percentage of dead cells in spheroids was averaged 4.6% ± 1.6% under all the conditions. This indicates that poor differentiation performance of spheroids under certain culture conditions was not caused by decreased cell viability.

In summary, these data demonstrate that smaller hPSC spheroids differentiate to DE cells more efficiently than bigger spheroids, and suspension culture promotes more effective DE differentiation than NFC hydrogel.

### Computational Simulation of Activin A Diffusion

**FIGURE 8:**
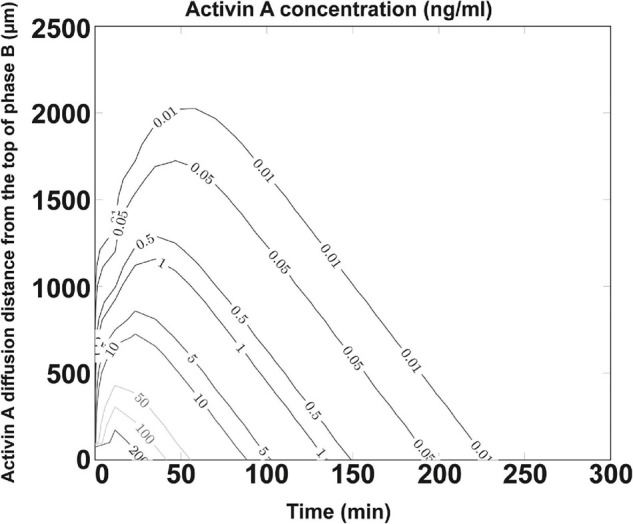
Concentration profile of activin A protein. Diffusion coefficient set as D_*Regression*_ = 7.31 × 10^– 7^ cm^2^/s for 26.0 kDa activin A protein and half-life λ = 20.3 min (slow estimation). Activin A was diffused from the interface between phase A and phase B into phase B. Phases A and B are illustrated in Supplementary Figure 1. *Y*-axis shows the activin A diffusion distance (μm) from the top of phase B. Activin A concentrations (ng/ml) at different positions in phase B during 250 min after the addition of the activin A-containing differentiation medium are shown by curves. Only the top 250 μm (8%) of the NFC hydrogel layer received 100 ng/ml activin A.

**FIGURE 9:**
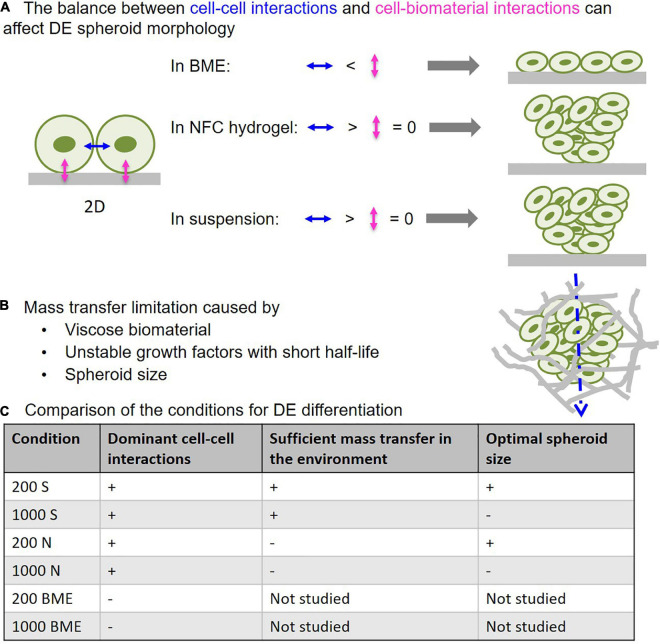
The critical influencing parameters in 3D DE differentiation. **(A)** Predominance of cell–biomaterial interactions in basement membrane extract (BME) leads to disintegration of 3D spheroid morphology, while predominance of cell–cell interactions in NFC hydrogel and suspension culture retains 3D spheroid morphology. **(B)** Viscose biomaterial and cell mass can limit mass transfer, thereby impairing differentiation that is mediated by growth factors with short half-lives. **(C)** Comparison of the studied 3D culture systems in terms of their properties affecting DE differentiation effectiveness.

Activin A used in the differentiation media is crucial for DE formation. Earlier studies showed that its effect is concentration-dependent, with high concentrations (100 ng/ml) specifying DE (D’Amour et al., 2005) and low concentrations specifying mesoderm (Schuldiner et al., 2000). The poor efficiency and effectiveness of DE formation from the bigger spheroids cultured in NFC hydrogel could indicate a poor diffusion of activin A due to mass transfer limitation caused either by cell masses in spheroids or by biomaterials. Simulating the diffusion of proteins within a spheroid is technically difficult to perform; thus, our model is focused on simulating the diffusion of activin A within the NFC hydrogel layer. By establishing a computational model based on Fick’s second law of diffusion and an NFC hydrogel-specific linear regression model based on the Einstein relation of kinetic theory and the viscous friction coefficient, we found that only the top 0.25 mm (8%) of the NFC hydrogel layer received 100 ng/ml activin A (Figure 8). Since protein half-lives in cell culture and in tissues are different (Rahman and Sadygov, 2017), our simulation using the *in vivo* half-life of activin A might overestimate or underestimate the activin A diffusion *in vitro*. Nonetheless, the simulation result indicates that activin A encountered considerable resistance to its transfer across NFC hydrogel, presumably due to hydrogel’s high viscosity (Bhattacharya et al., 2012). The limited mass transfer may explain the low effectiveness of the DE differentiation in NFC hydrogel (Figure 9).

## Discussion

To overcome the major challenges in spheroid and organoid technology and increase the effectiveness of stem cell differentiation in 3D environments, we examined how 3D cell culture systems and spheroid size influence DE differentiation of human iPSCs. Here we show that suspension culture can effectively generate DE cells from 3D human iPSC spheroids, whereas the use of biomaterials in DE differentiation may cause issues with mass transfer and spheroid formation. These findings are summarized in a schematic diagram (Figure 9). BME, a biomaterial with cell-interacting properties, produced strong cell–matrix interactions that caused 3D spheroids to spread out into monolayers (Figure 9). NFC hydrogel, a biomaterial with poor cell-interacting properties, was able to maintain 3D spheroid morphology during DE differentiation (Figure 9). However, NFC hydrogel impeded growth factor diffusion presumably due to its high viscosity, and therefore DE formation was less effective than in suspension culture. We also found an inverse correlation between spheroid size and the effectiveness of DE formation; smaller spheroids (with initial 200 cells per spheroid) differentiated more effectively than the larger ones (with initial 1,000 cells per spheroid). In addition, bigger spheroids (with initial 200, 500, and 1,000 cells per spheroid) formed stable cell aggregates after 24 h, while spheroids that are too small (with initial 50 or 100 cells per spheroid) did not retain their 3D morphology in suspension or in NFC hydrogel possibly due to poor cell–cell interactions. The importance of cell–cell interaction in the formation of dermal fibroblast spheroids has recently been demonstrated by using a micropatterned hydrogel (Kim et al., 2019).

Mass transfer, also known as mass transport, is important for successful 3D cell culture and tissue engineering (Antoni et al., 2015). Sufficient mass transfer ensures the proper supply of nutrients and regulatory factors to cells and therefore generates desired cell and tissue products. Insufficient mass transfer can be caused by the presence of biomaterials and cells. Mass transfer is particularly critical when growth factors are involved because many growth factors have short half-lives, and delayed delivery to target cells results in reduced dosage to cells. By establishing a computational model to simulate growth factor diffusion, we found that activin A poorly diffused across NFC hydrogel, which may be due to the short half-life of activin A and the high viscosity of NFC hydrogel. This problem may be common for all 3D cell culture systems combining growth factors with short half-lives and highly viscose biomaterials. This issue may be solved by stabilizing growth factors or increasing the permeability of NFC hydrogel. The half-life of Wnt-3a increases when using liposomal packaging (Dhamdhere et al., 2014), so similar approaches may be applicable for activin A. However, liposomal packaging increases the radii of said growth factors, potentially hindering diffusion. Fortunately, the effect of such packaging could be predicted with the computational model described in this study. Replacing growth factors with stable small molecules is another solution. We have previously tested IDE1 as a substitute for activin A in DE differentiation, but unfortunately, it was ineffective (Bogacheva et al., 2018). Another study tested several small molecules in DE differentiation but still did not find an equally effective chemical to replace activin A (Tasnim et al., 2015). A high-content screening study identified two ROCK inhibitors as DE inducers in human and mouse ESCs (Korostylev et al., 2017). However, the undifferentiated ESCs used in their study were positive for DE markers, which may indicate intrinsic bias of the DE marker-positive ESC population toward endoderm differentiation as shown in an earlier study (Allison et al., 2018). Hydrogel permeability is an intrinsic property. Therefore, increasing permeability requires extensive investigation and may involve undesired changes in other hydrogel properties.

Basement membrane extract, representing another class of biomaterials that have cell-interacting properties, was also used in this study. The finding that BME could not maintain 3D spheroid morphology during DE differentiation is interesting. We speculate that this was the result of the imbalance between cell–matrix interactions and cell–cell interactions (Figure 9). It is known that undifferentiated ESCs and iPSCs can interact with BME (Xu et al., 2001). Upon embedding iPSC spheroids in BME, strong cell–BME interactions may override cell-cell interactions, thereby causing disruption of spheroid morphology. Decreasing E-cadherin expression during DE differentiation (D’Amour et al., 2005) further reduces cell–cell interactions, thereby contributing to disrupted spheroid morphology. In contrast, NFC hydrogel has negligible adhesion forces to stem cells (Harjumaki et al., 2019)—minimizing cell–matrix interactions. Thus, the predominance of cell–cell interactions may account for the well maintenance of the spheroid shape in NFC hydrogel (Figure 9). These conclusions are supported by Nie et al. (2020) who previously showed with human keratinocytes that decreased cell-substrate adhesion was the main driving force in the spheroid formation and at the same time cell–cell interaction forces increased and exceeded cell–biomaterial interaction force levels.

The migrating cells in BME are presumably DE cells as shown by the immunofluorescence of SOX17 (data not shown). The reason for their death from day 4 onward could be due to the nonsupportive environment. Although DE cells can be derived from human PSCs on BME in 2D culture, DE cells, in fact, have limited ability to attach to BME as shown by the downregulation of laminin 111-specific integrins in DE cells (Kanninen et al., 2016a) and failure on re-attachment to BME after detachment (Kanninen et al., 2016b). Derivation of DE cells on BME in 2D culture may involve an undifferentiated PSC-produced niche, which requires further investigation.

Suspension culture is a biomaterial/scaffold-free system, meaning that there is no potential mass transfer limitation caused by biomaterials. In some cases, cells are attached to floating microcarriers. Nonetheless, all suspension cultures ensure equal supply to all spheroids. Another biomaterial/scaffold-free system that has been used in spheroid technology is the hanging-drop system. It has been used to form spheroids of dermal papilla cells in a controllable and scalable manner (Lin et al., 2016). By performing DE differentiation that involves the use of growth factor, we clearly demonstrate that suspension culture is a superior 3D culture system to biomaterial-based 3D systems because it provides equal molecular diffusion among all the spheroids, and therefore produces efficient DE differentiation. Suspension culture is scalable (Amit et al., 2010; Li et al., 2018) and can be performed in a bioreactor with tight control of cell culture conditions for mass production (Lock and Tzanakakis, 2009). Unlike biomaterial-based culture systems, suspension culture does not provide physical constraint and therefore spheroids grew faster, as shown by more significant increases in diameter in suspension culture than in NFC hydrogel culture (Supplementary Figures 4, 6).

In addition to biomaterials, cells can also limit mass transfer. Earlier studies have found that hepatocyte spheroids with diameters of more than 100 μm (Glicklis et al., 2004) or human ESC and iPSC spheroids with diameters of more than 350 μm (Amit et al., 2010) had cell viability rates below 90%. Our 4-day protocol for DE differentiation generated more than 90% viable cells (Supplementary Figure 13) in spheroids with the average diameters below 350 μm (Figure 3B and Supplementary Figure 5) in all the conditions studied. Despite the high cell viability, we found that iPSC spheroids with a higher cell number had lower differentiation effectiveness. Our finding agrees with an earlier report showing that large spheroids generated fewer SOX17- and HNF3B-positive cells (Farzaneh et al., 2018). The low effectiveness of differentiation in large spheroids is presumably due to the insufficient supply of growth factors. Computational simulation of growth factor diffusion through spheroids is not straightforward because growth factor can bind to any cells on its route of diffusion. There have been computational models for oxygen permeation into a spheroid (Aleksandrova et al., 2016; Grimes and Currell, 2018), and more comprehensive models could be established to estimate protein diffusion in a cell spheroid with consideration of growth factor-receptor binding and receptor abundance. Although smaller spheroids ensure a sufficient supply of growth factor, they may not survive during DE differentiation, as shown in the current study. It is known that cadherins mediate homophilic binding between the same types of cells. DE cells express a lower level of E-cadherin and a higher level of N-cadherin than undifferentiated ESCs and iPSCs (D’Amour et al., 2005). During DE differentiation, weaker cadherin-mediated interactions could have been generated between undifferentiated PSCs and differentiating cells leading to the failure of smaller spheroids to retain their 3D morphology.

We found that cell viability inside the spheroids can be improved with the constant presence of the ROCK inhibitor Y-23720 in the differentiation media. An earlier study found a priming effect of a high concentration of Y-23720 on mesendoderm differentiation (Maldonado et al., 2016); to ensure the use of Y-23720 does not have a negative effect on DE differentiation, we analyzed its influence on the gene expression of specific pluripotency, DE, mesendoderm, and ectoderm markers. Among the three studied DE markers, only *CER1* was significantly altered by 4.2-folds by the 7-day treatment with 10 μM Y-23720 (Supplementary Figure 2). Cer1 is a secreted protein participating in the regulation of Nodal, Wnt, and BMP signaling pathways and is a marker of the endoderm specification (Iwashita et al., 2013). However, the 4.2-fold increase is much less than the 30,000-fold increase observed during the directed DE differentiation (Figure 4E). Based on our data, the effect of Y-23720 on the DE differentiation of human PSCs was relatively mild, but its biological impact needs further investigation.

Several studies have demonstrated that 3D cell culture systems can produce DE cells from human ESCs and iPSCs. A small molecule IDE1 was shown to induce DE differentiation of human iPSCs in a poly(ε-caprolactone)-based scaffold, but the expression of *HNF3B* and *SOX17* was only induced no more than 20-folds (Hoveizi et al., 2014). In contrast to 3D culture, IDE1 (Bogacheva et al., 2018) and its analog IDE2 (Tasnim et al., 2015) were found to be ineffective in 2D DE differentiation. Another study produced DE cells in alginate hydrogel using activin A and Wnt-3a with 800- and 300-fold induction of *HNF3B* and *SOX17*, respectively (Chayosumrit et al., 2010), whereas we observed more than 4000- and 40,000-fold increase in the expression of *HNF3B* and *SOX17*, respectively. A recent study showed that 3D DE differentiation in suspension culture has an increased proliferation coefficient and higher speed of the upregulation of DE markers in comparison with adherent culture (Yabe et al., 2019). Another study successfully produced DE aggregates in chemically defined, xeno-free suspension culture, which was demonstrated to be scalable by using bioreactors (Sahabian et al., 2019). In addition, 3D culture has been proven to improve the maturation of hepatic cells (Miki et al., 2011; Freyer et al., 2016) and pancreatic cells (Wang and Ye, 2009).

We found that the remaining problem with NFC hydrogel culture is high viscosity. Utilization of chemically stable small molecules to replace growth factors would potentially solve mass transfer limitation and thus would allow more effective use of NFC hydrogel in stem cell differentiation and spheroid formation. The fast growth kinetic in suspension culture might yield larger spheroids that can inhibit mass transfer. Dissociating spheroids followed by making smaller spheroids, for example 200 cells per spheroid, would be a strategy to maintain sufficient mass transfer during differentiation experiments.

This study demonstrates that the spheroids with initial 200 cells per spheroid in suspension culture can efficiently produce DE cells (Figure 9C). However, further work is required to quantitatively demonstrate the inverted correlation between the size of the spheroids and the expression of nuclear DE markers, which was not demonstrated in the current study due to technical limitations in flow cytometry detecting intracellular proteins. Moreover, it is necessary to assess the potential of these cells in further differentiation into endoderm derivatives, such as hepatic, pancreatic, and intestinal cells. To further differentiate DE spheroids generated in the current study, we could continue using suspension culture system to ensure sufficient mass transfer. If stable small molecules to replace growth factors are available, hydrogel-based 3D culture systems can also be utilized to provide physical support and constraint.

## Conclusion

In the current study, we show how the size of the human iPSC spheroids and 3D culture conditions influence DE differentiation. We found that the ROCK inhibitor improves human iPSC spheroid viability and can be applied for the entire length of DE differentiation. The spheroid size determines the availability of growth factors as well as nutrients and oxygen supply to all the cells. Suspension culture provides sufficient mass transfer and thus generates more effective DE differentiation than NFC hydrogel-based culture. When using biomaterials, cell–matrix interaction and mass transfer should be considered because they can affect 3D cell spheroid morphology and the effectiveness of growth factor-mediated differentiation, respectively. Our findings are beneficial for the development of human iPSC-derived 3D cell models, which have applications in drug research field for the evaluation of toxicity and efficacy of drug candidates, developmental biology studies, and regenerative medicine.

## Data Availability Statement

The original contributions presented in the study are included in the article/Supplementary Material, further inquiries can be directed to the corresponding author/s.

## Author Contributions

Y-RL conceived, designed, and supervised the research, carried out some of the experiments presented in the manuscript, analyzed the data, and wrote the Introduction, Results, and Discussion sections of the manuscript. MSB carried out most of the experiments presented in the manuscript, analyzed most of the data, made the figures, and wrote the Materials and Methods section and part of the Results section. RH performed preliminary DE differentiation of human PSC spheroids without spheroid size control in NFC hydrogel and suspension culture (data not shown). EF carried out some of the experiments presented in the manuscript, measured spheroid diameters, and analyzed qPCR data. AT performed preliminary DE differentiation of human PSC spheroids without spheroid size control in NFC hydrogel and suspension culture (data not shown), performed computational simulation of activin A diffusion, and wrote the modeling part of the Methods section. MAB helped in some experiments and measured spheroid diameters. AL provided valuable advice during research planning, analyzed qPCR data, and edited the manuscript. All authors commented on the final version of the manuscript.

## Conflict of Interest

The authors declare that the research was conducted in the absence of any commercial or financial relationships that could be construed as a potential conflict of interest.

## Publisher’s Note

All claims expressed in this article are solely those of the authors and do not necessarily represent those of their affiliated organizations, or those of the publisher, the editors and the reviewers. Any product that may be evaluated in this article, or claim that may be made by its manufacturer, is not guaranteed or endorsed by the publisher.
