# Supplementary material for: Differentiation of Human Pluripotent Stem Cells Into Definitive Endoderm Cells in Various Flexible Three-Dimensional Cell Culture Systems: Possibilities and Limitations
Source: Front Cell Dev Biol. 2021 Sep 9;9:726499. doi: 10.3389/fcell.2021.726499 (PMC8459831; doi:10.3389/fcell.2021.726499)
Supplement: Supplementary file 1 [file Data_Sheet_1.PDF]

Differentiation of human pluripotent stem cells into definitive endoderm cells in various flexible three-dimensional cell culture systems: possibilities and limitations

Mariia S. Bogacheva[1,4], Riina Harjumäki[1,4], Emilia Flander[1,5], Ara Taalas[1,5], Margarita A. Bystriakova[1], Marjo Yliperttula[1], Xiaoqiang Xiang[2,*], Alan W. Leung[3], Yan-Ru Lou[2,1,6,*]

*Lead contact and corresponding author: Yan-Ru Lou, email: yanru_lou@fudan.edu.cn

Supplementary material

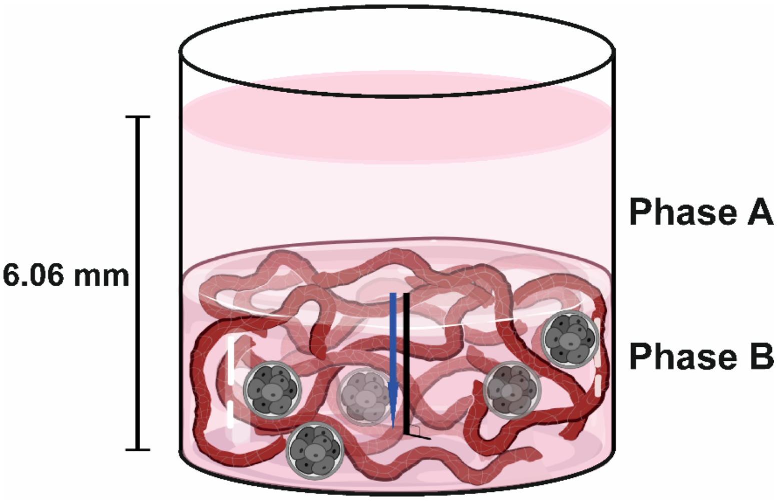

Figure S1. A cell culture well containing spheroids in NFC hydrogel. Phase A contains 100 µl of the medium. Phase B contains 3D cell spheroids embedded in 100 µl of NFC hydrogel diluted in the medium. The blue arrow indicates the direction of activin A diffusion within the NFC hydrogel layer.

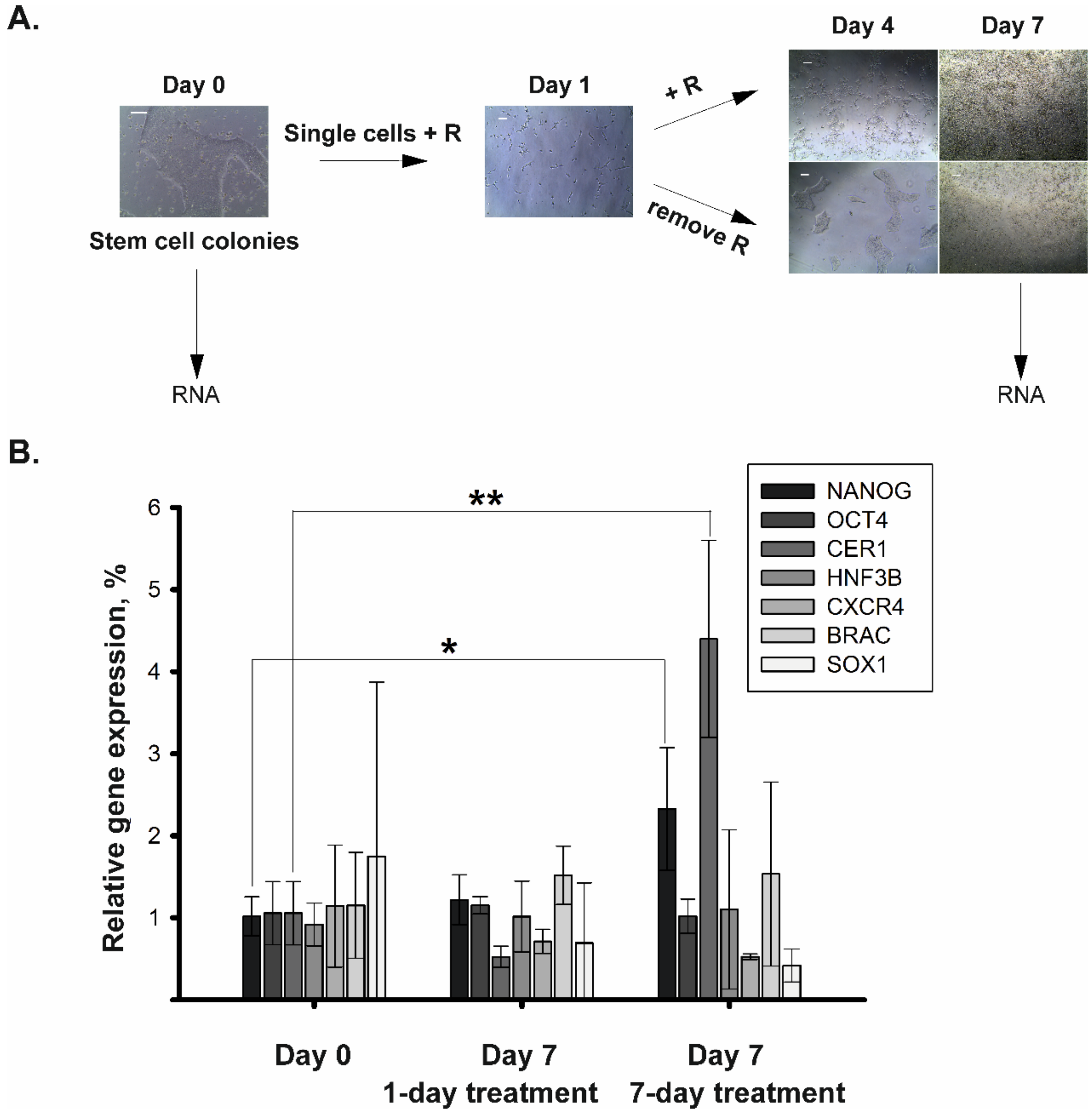

Figure S2. The effect of the ROCK inhibitor Y-27632 (R). (A) Schematic of the experimental setup. iPS(IMR90)-4 cells grown in colonies (day 0) were dissociated into single cells, treated with 10 µM Y-27632 and cultured in 2D culture condition in mTeSR™1 medium on Matrigel™-coated wells on the first day. Then cells were either cultured in mTeSR™1 medium (remove R) or mTeSR™1 medium with 10 µM Y-27632 (+R) for 6 days. RNA for qPCR analysis was collected from untreated SC colonies (day 0) and cells after 7 days of culture with and without Y-27632. Scale bars = 100 µm. (B) Expression profiles of SC (*OCT4* and *NANOG*), DE (*CER1, HNF3B,* and *CXCR4*), mesendoderm (*BRACHYURY (BRAC)*), and ectoderm (*SOX1*) mRNA during 7 days of stem cell culture with 1-day and 7-day Y-27632 exposure. The mRNA expression was measured by real-time qPCR. Relative mRNA expression was normalized with housekeeping gene *RPLP0*, and fold inductions were calculated with the reference to the untreated iPS(IMR90)-4 cells at day 0. N = 3 biological samples. Error bars are SD. Data were analyzed by one-way ANOVA followed by Sidak's multiple comparisons test. * adjusted $P < 0.05$ and ** adjusted $P < 0.01$ are shown above lines.

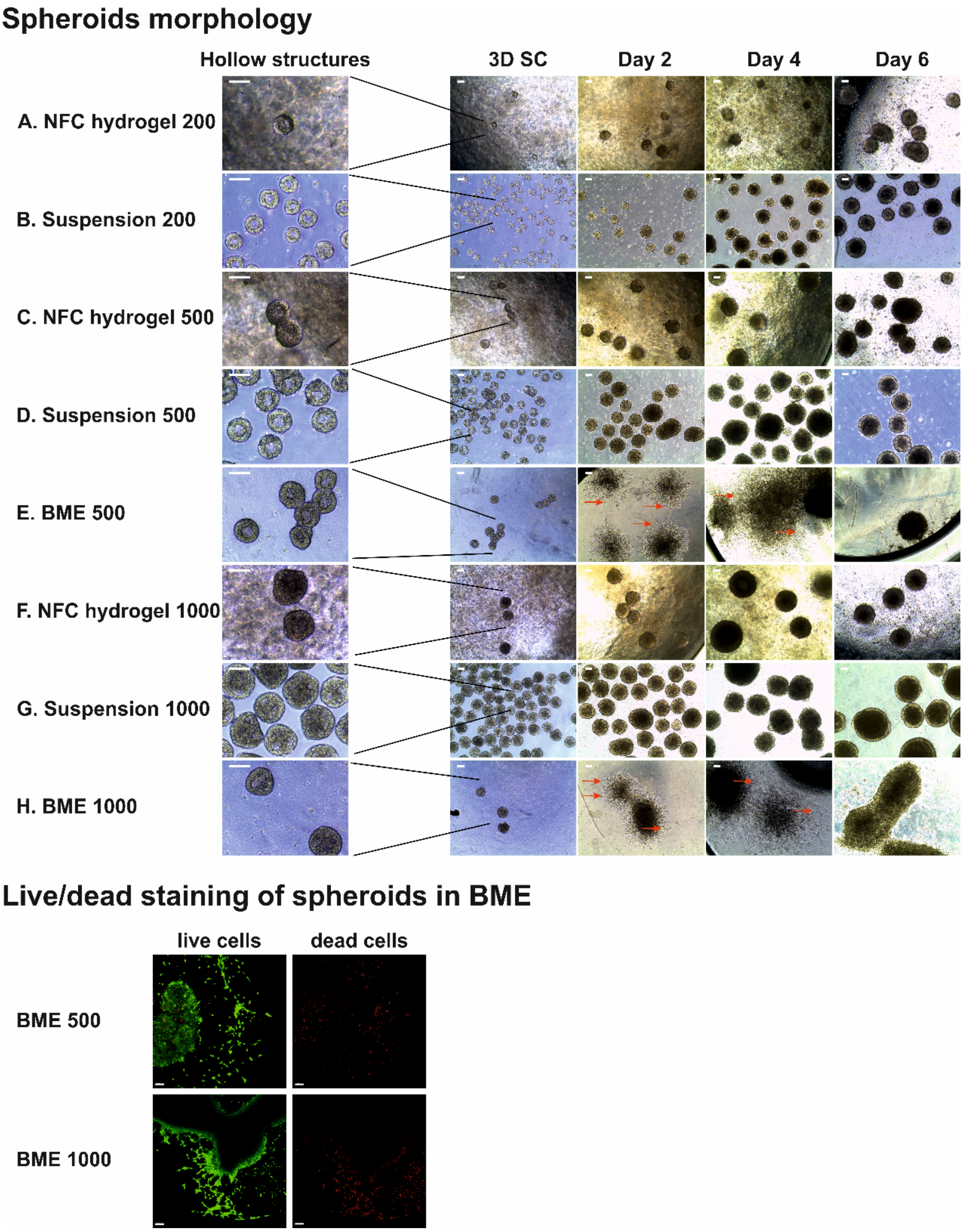

Figure S3. Morphology and live/dead staining of iPS(IMR90)-4 cell spheroids. Spheroids with the initial cell number of 200, 500, and 1000 cells per spheroid at the undifferentiated stage (3D SC) and at days 2, 4, and 6 of DE differentiation in NFC hydrogel (A, C, and F), suspension (B, D, and G), and BME (E and H). ROCK inhibitor Y-27632 at 10 μM was used in the differentiation medium during the entire experiment. Images showing hollow structures are zoomed in from the images at 3D SC stage. Arrows in the images E and H point out the cells at the 2D areas, exhibiting typical DE morphology in 2D culture. Spheroids at day 6 of DE differentiated in BME were stained using a Live/dead viability/cytotoxicity kit. Cells that migrated outside spheroids were stained in green (live cells) or red (dead cells). Scale bars = 100 μm.

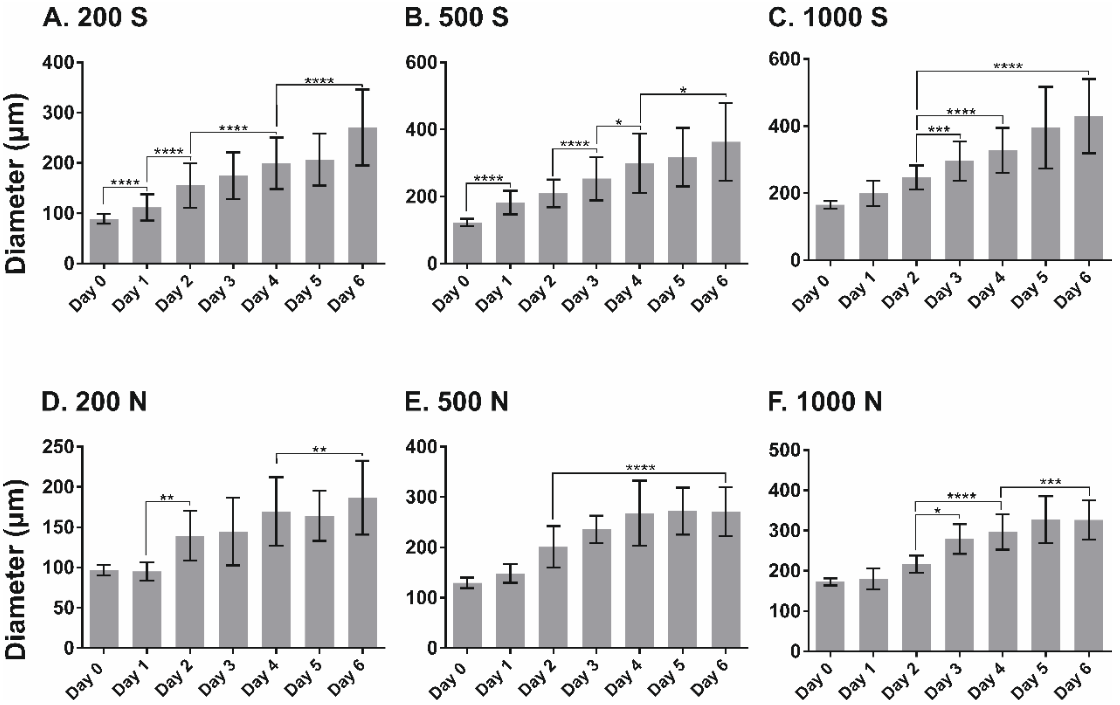

Figure S4. Spheroid sizes during differentiation. Diameters (µm ± SD) of iPS(IMR90)-4 spheroids with the initial cell number of 200, 500, and 1000 cells per spheroid in suspension, named 200S (A), 500S (B), and 1000S (C), and in NFC hydrogel, named 200N (D), 500N (E), and 1000N (F), respectively are shown as a function of time (day). Because a normality test indicates that the data were not sampled from a Gaussian population, Kruskal-Wallis test followed by Dunn's multiple comparisons test was used. Statistical significance * adjusted $P < 0.05$, *** adjusted $P < 0.001$, and **** adjusted $P < 0.0001$ are shown above lines.

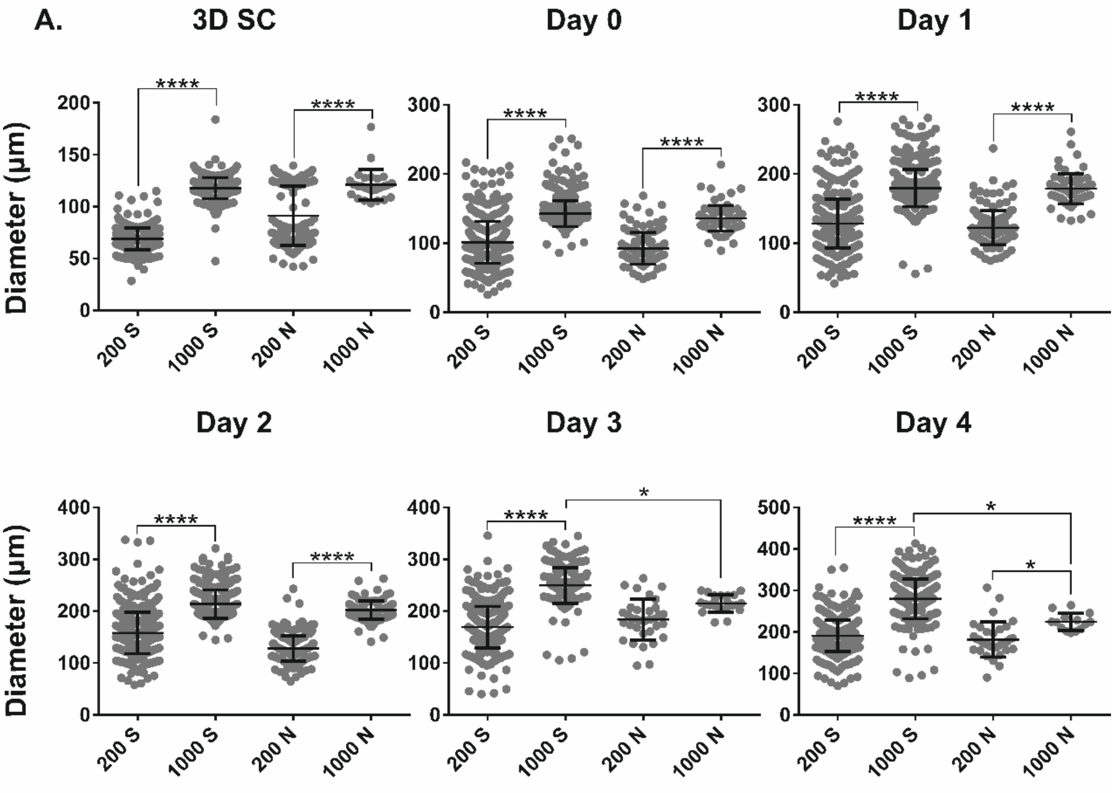

B.

Condition/ Timepoint	200S	1000S	200N	1000N
**3D SC**	68.79 ± 10.56	117.7 ± 10.2	91.19 ± 28.75	121.2 ± 14.92
**Day 0**	101.1 ± 30.23	142.9 ± 18.7	92.54 ± 22.84	136 ± 18.37
**Day 1**	128.7 ± 35.23	179.7 ± 26.81	122.3 ± 24.65	178.7 ± 21.88
**Day 2**	158.1 ± 40.06	213.8 ± 27.43	128.3 ± 24.51	202.2 ± 17.67
**Day 3**	169.4 ± 39.9	249.7 ± 34.45	184 ± 39.19	215 ± 16.41
**Day 4**	190.8 ± 38.26	279.6 ± 47.86	181.7 ± 42.27	224.2 ± 20.74

Figure S5. Spheroid sizes during differentiation. (A) Size distribution of GM23720B cell spheroids with the initial cell number of 200 and 1000 cells per spheroids during DE differentiation in suspension (S) and NFC hydrogel (N). Diameters (µm) were measured daily during the experiment. Horizontal lines are mean values, and vertical lines are SD. Because a normality test indicates that the data were not sampled from a Gaussian population, Kruskal-Wallis test followed by Dunn's multiple comparisons test was used. Statistical significance * adjusted $P < 0.05$ and **** adjusted $P < 0.0001$ are shown above lines. (B) Average diameter of spheroids with the initial cell number of 200 and 1000 cells per spheroid (µm) ± SD at each day of the DE differentiation of GM23720B cells.

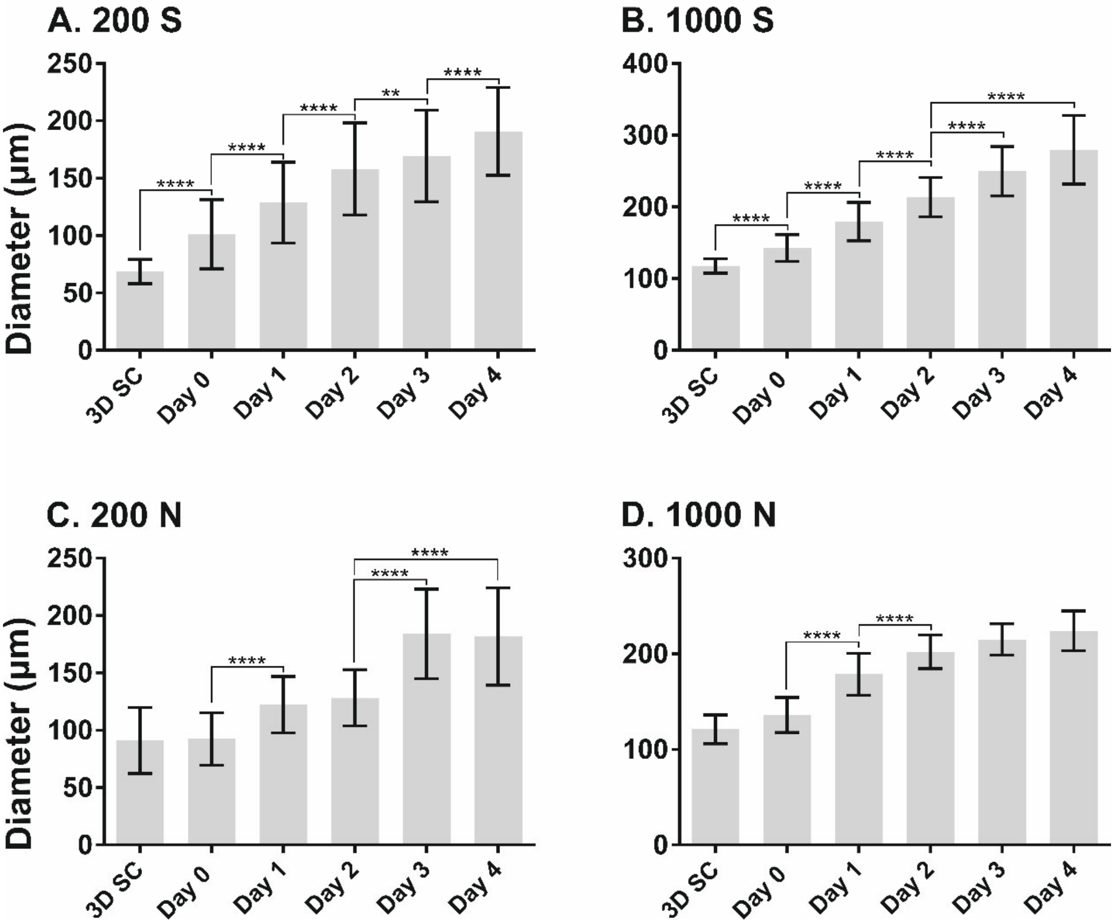

Figure S6. Spheroid sizes during differentiation. Diameters (µm ± SD) of GM23720B spheroids with the initial cell number of 200 and 1000 cells per spheroid in suspension, named 200S (A) and 1000S (B), and in NFC hydrogel, named 200N (C) and 1000N (D), respectively are shown as a function of time (day). Because a normality test indicates that the data were not sampled from a Gaussian population, Kruskal-Wallis test followed by Dunn's multiple comparisons test was used. Statistical significance ** adjusted $P < 0.01$ and **** adjusted $P < 0.0001$ are shown above lines.

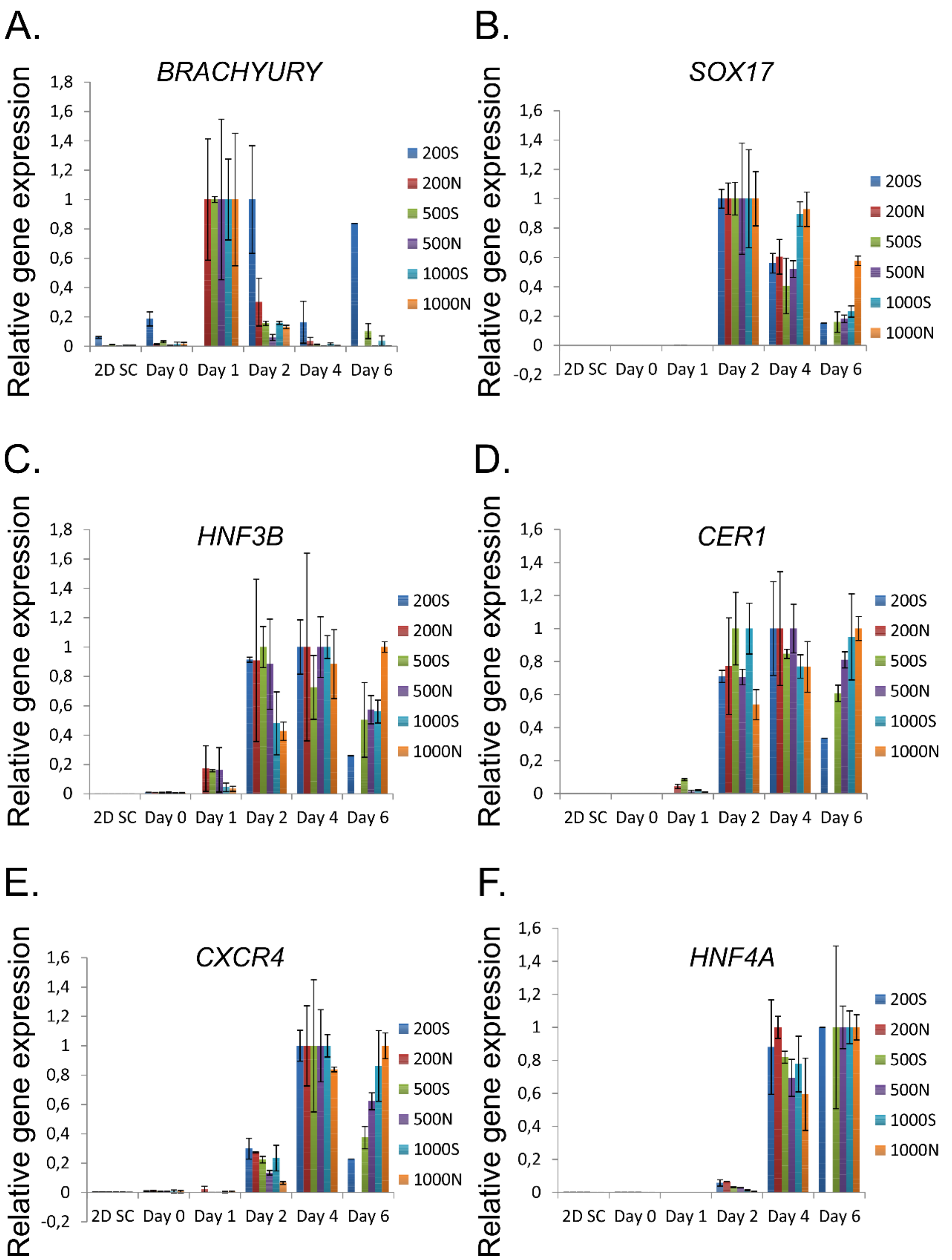

Figure S7. The mRNA expression patterns of the mesendoderm (*BRACHYURY*), DE (*SOX17*, *HNF3B*, *CER1*, and *CXCR4*), and hepatic endoderm (*HNF4A*) specific markers during DE differentiation of iPS(IMR90)-4 cells in 3D spheroids with the initial cell number of 200, 500, and 1000 cells per spheroid in suspension (S) and NFC hydrogel (N). The same data shown in Fig. 4 are normalized to the highest level during 3D DE differentiation. Under most conditions *BRACHYURY* was induced and peaked at day 1 (A), followed by *SOX17*, which was induced and peaked at day 2 (B). As *SOX17* expression indicates DE cell specification, day 2 appeared to be the stage when DE was specified for differentiating cells under most conditions (B-F). As expected,

the expression of two other more downstream DE markers *HNF3B* and *CER1* was also dramatically induced at day 2 but peaked at a later time point than *SOX17* between day 2 and day 4 (C-D). Another DE marker *CXCR4* was induced at day 2 but peaked only at around day 4 (E). And finally, the expression of the late DE marker *HNF4A* peaked between day 4 and day 6 (F). In summary, the gene expression follows the sequence *BRACHYURY* (day 1) → *SOX17* (day 2) → *HNF3B*/*CER1* (days 2-4) → *CXCR4* (day 4) → *HNF4A* (day 6), which confirms other previous studies.

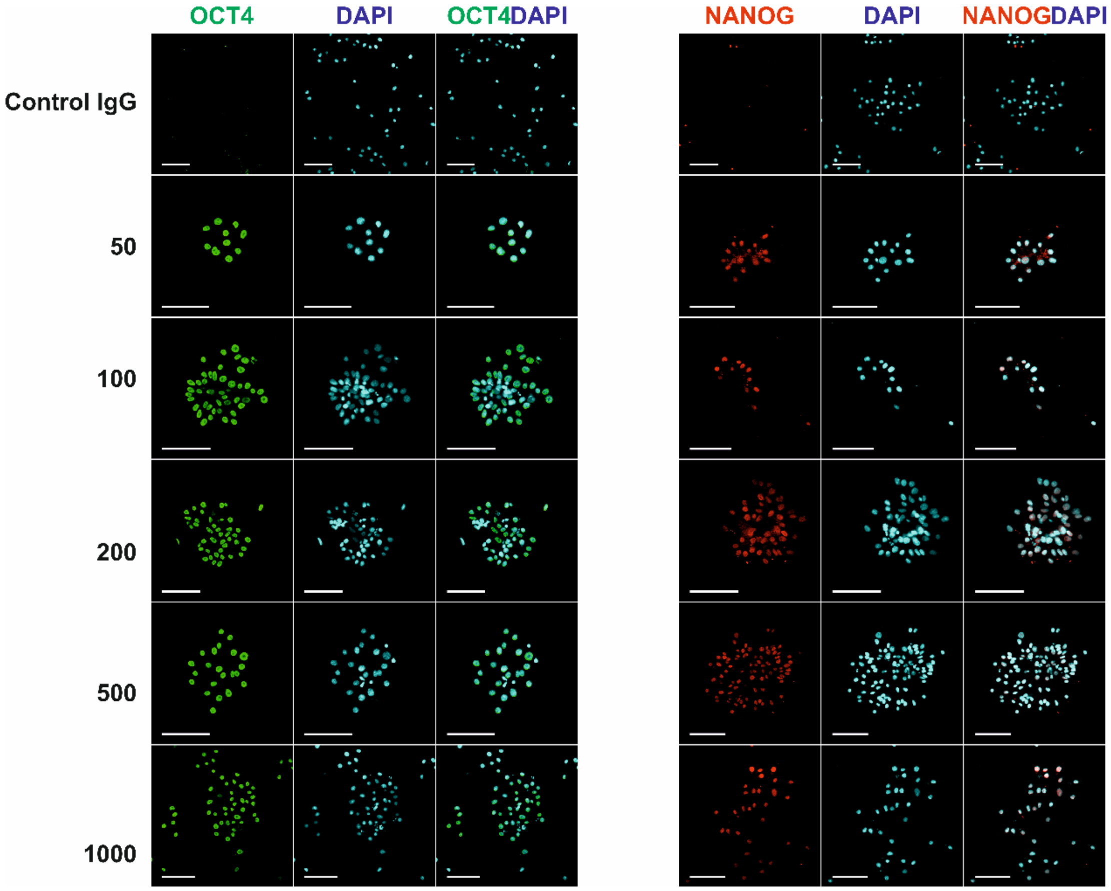

Figure S8. Expression of OCT4 and NANOG proteins in undifferentiated 3D iPS(IMR90)-4 cell spheroids. The iPS(IMR90)-4 cell spheroids were formed in AggreWell™400 plate for 24 hours and were dissociated with Accutase™. Single cells were cultured in mTeSR™1 supplemented with 10 µM Y-27632 in laminin-521 coated well plate. Cells were fixed three hours later. Nuclei of cells were stained with DAPI (blue). Proteins of interest were stained either with Alexa Fluor 488 (OCT4), showed in green, or with Alexa Fluor 594 (NANOG), showed in red. Scale bars = 100 µm.

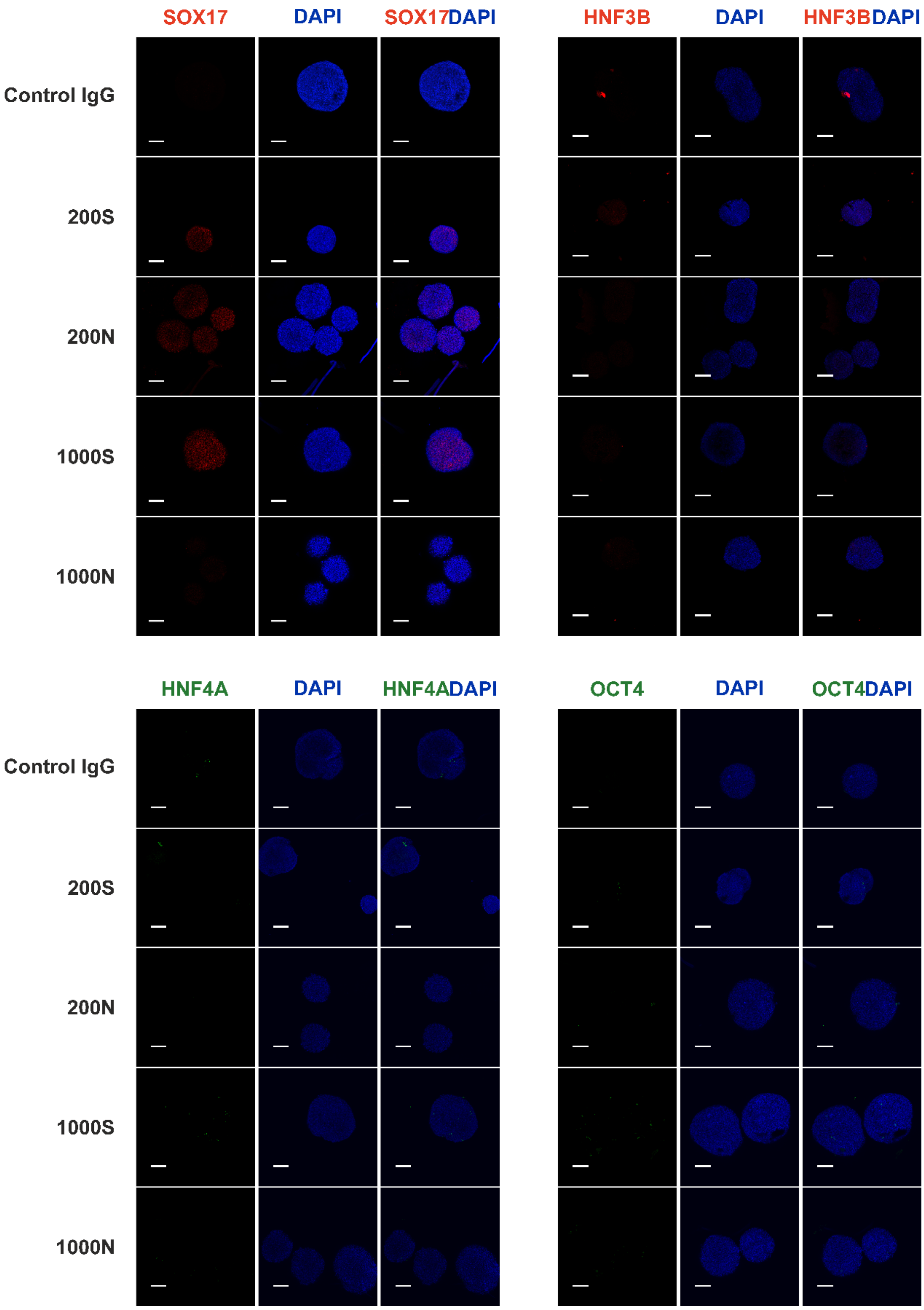

Figure S9. Expression of SOX17, HNF3B, HNF4A, and OCT4 proteins in GM23720B cell spheroids at day 4 of 3D DE differentiation in suspension (S) or NFC hydrogel (N). Nuclei of cells were stained with DAPI (blue). Proteins of interest were stained either with Alexa Fluor 488 (HNF4A and OCT4), showed in green, or with Alexa Fluor 594 (SOX17 and HNF3B), showed in red. Scale bars = 100 µm.

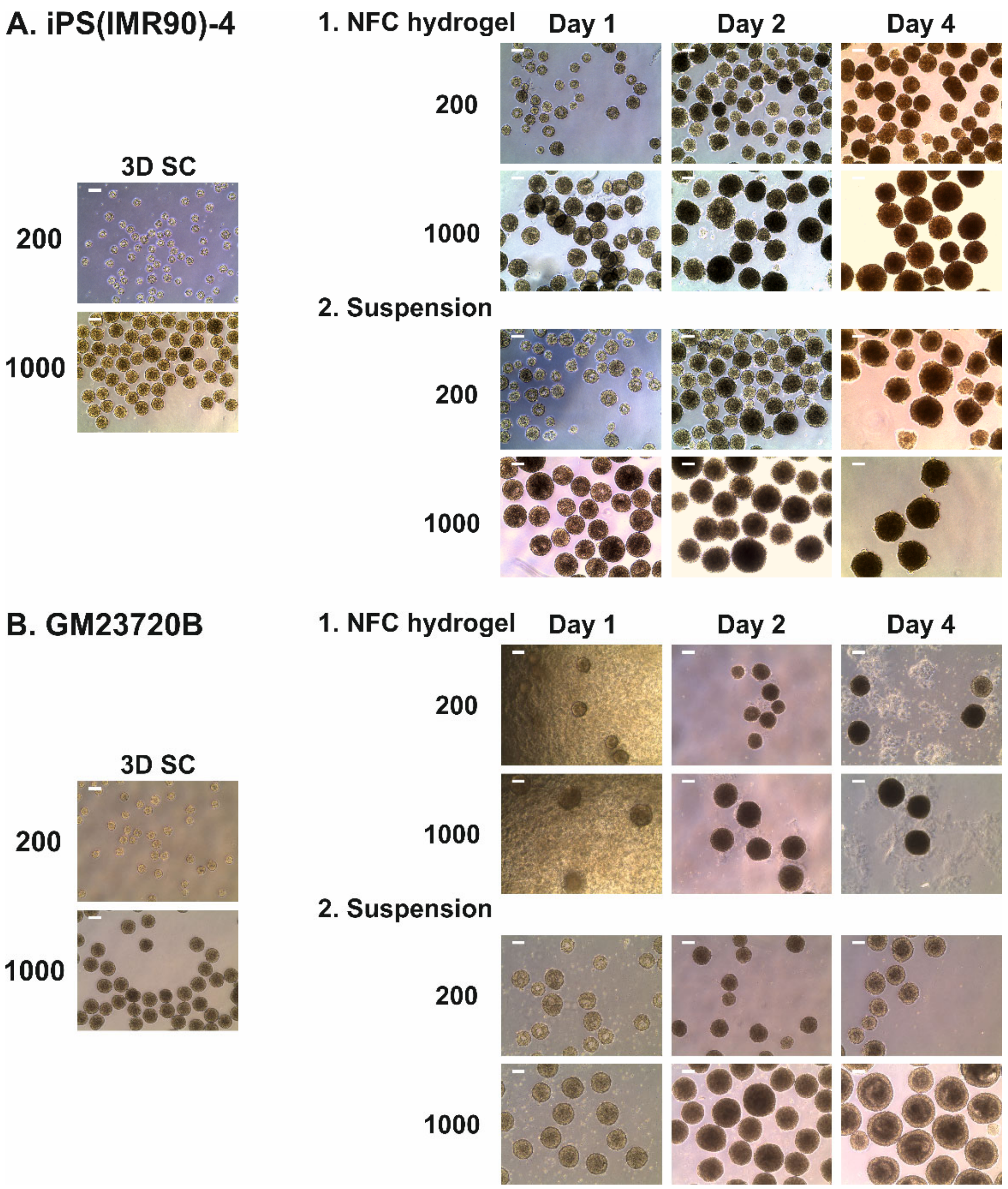

Figure S10. Morphology of iPS(IMR90)-4 (A) and GM23720B (B) cell spheroids with the initial cell number of 200 and 1000 cells per spheroid at the undifferentiated stage (3D SC) and at days 1, 2, and 4 of DE differentiation in suspension and NFC hydrogel. Scale bars = 100 µm.

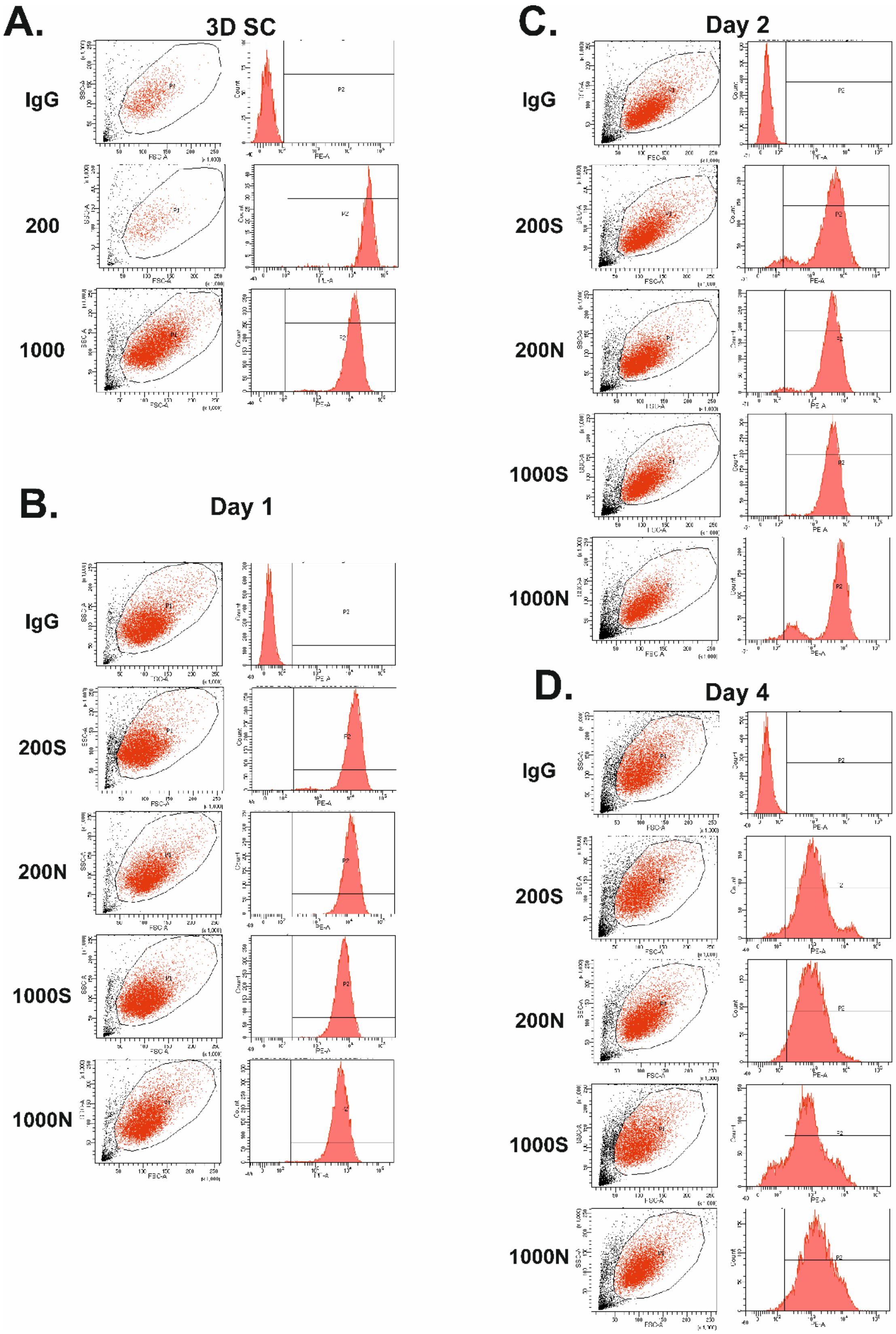

Figure S11. Unprocessed data of the dynamic SSEA4 expression in iPS(IMR90)-4 cells and their derivatives during DE differentiation of 3D spheroids with the initial cell number of 200 and 1000 cells per spheroid in suspension (S) and NFC hydrogel (N) utilized for the Figure 6A. (A) SSEA4 protein expression at the stage of undifferentiated cells (3D SC) and (B) day 1, (C) day 2, and (D) day 4 in differentiation experiments.

Isotype control (IgG) was used for the determining of SSEA4^{-} population (P2). This experiment was performed three times, and only one of the experimental results is shown here.

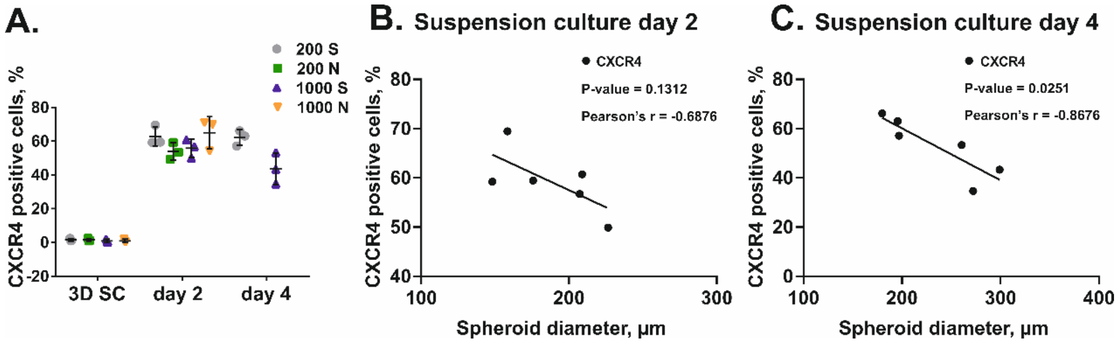

Figure S12. The quantitative expression of markers and the correlation between spheroid diameters and the effectiveness of the DE differentiation in GM23720B cells. (A) CXCR4 protein expression pattern in GM23720B cell spheroids (3D SC) and their derivatives during 3D DE differentiation in suspension (S) and NFC hydrogel (N). N = 3 biological repeats. (B) Pearson correlation between spheroid diameters and percentages of live CXCR4$^+$ cells at day 2 of the 3D DE differentiation in suspension. No statistically significant Pearson correlation is detected. (C) Pearson correlation between spheroid diameters and percentages of live CXCR4$^+$ cells at day 4 of the 3D DE differentiation in suspension. A statistically significant Pearson correlation is detected ($P = 0.0251$).

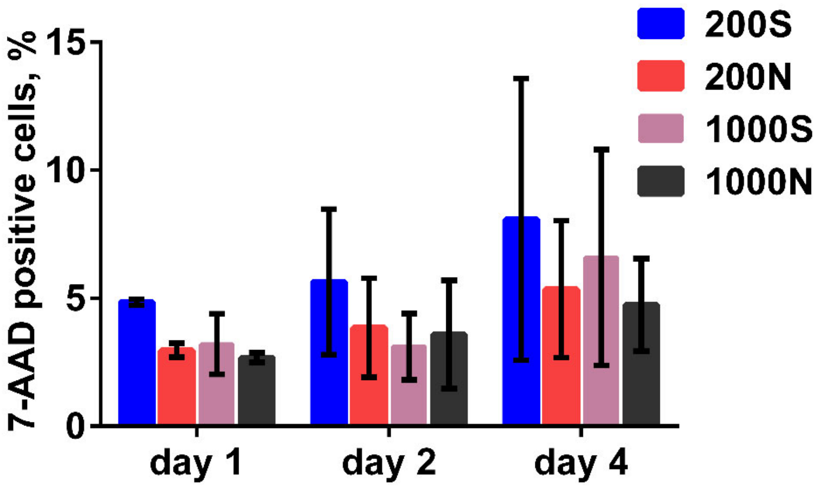

Figure S13. Cell death of iPS(IMR90)-4 cell spheroids with the initial cell number of 200 and 1000 cells per spheroid during DE differentiation (day 1, day 2, and day 4) in suspension (S) and NFC hydrogel (N). The graph shows the percentage (mean ± SD, n = 4 biological samples) of dead cells stained with 7-AAD fluorescent stain and detected in the PE-Cy5 channel by flow cytometry analysis. One-way ANOVA followed by Tukey's multiple comparisons test found no statistically significant difference between any pairs.

Table S1. Primers and TaqMan® Gene Expression Assay mixes for qPCR

Gene	Accession	Size (bp)	Sequence (5′ to 3′)
RPLP0	NM_001002.3<br>NM_053275.3	74	F: AATCTCCAGGGGCACCATT<br>R: CGCTGGCTCCCACTTTGT
OCT4	NM_002701.4<br>NM_203289.4<br>NM_001173531.1	161	F: CAGTGCCCGAAACCCACAC<br>R: GGAGACCCAGCAGCCTCAAA
NANOG	NM_024865.2	80	F: GCAGAAGGCCTCAGCACCTA<br>R: GGTTCCCAGTCGGGTTCAC
HNF3B	NM_021784.4<br>NM_153675.2	89	F: GGGAGCGGTGAAGATGGA<br>R: TCATGTTGCTCACGGAGGAGTA
SOX17	NM_022454.3	51	F: CAGAATCCAGACCTGCACAA<br>R: CGACTTGCCCAGCATCTT
BRACHYURY	NM_001270484.1<br>NM_003181.3	118	F: AGAACGGCAGGAGGATGTTTCC<br>R: ACGTACTTCCAGCGGTGGTTGT
**Gene**	**TaqMan® Gene Expression ID**		
RPLP0	Hs99999902_m1		
CER1	Hs00193796_m1		
CXCR4	Hs00607978_s1		
SOX1	Hs01057642_s1		
HNF4A	Hs00230853_m1		

Table S2. Antibodies used in immunofluorescent staining

**Antibody**	**Manufacturer**	**RRID**	**Dilution ratio**
Rabbit anti-OCT4	Santa Cruz Biotechnology, sc-9081	AB_2167703	1:250 and 1:500
Goat anti-NANOG	R&D Systems, AF1997	AB_355097	1:50
Rabbit anti-HNF4A	Sigma–Aldrich, HPA004712	AB_1079075	1:45
Goat anti-SOX17	R&D Systems, AF1924	AB_355060	1:50
Goat anti-HNF3B	Santa Cruz Biotechnology, sc-6554	AB_2262810	1:25
Normal rabbit IgG	Peprotech, 500-P00	AB_2722620	1:450, 1:1250, and 1:2500
Normal goat IgG	Peprotech, 500-G00	-	1:50 and 1:100
Donkey anti-goat IgG Alexa Fluor 594	Invitrogen, A11058	-	1:200
Goat anti-rabbit IgG Alexa Fluor 488	Life Technologies, A11008	-	1:200
